# An approach of gene regulatory network construction using mixed entropy optimizing context-related likelihood mutual information

**DOI:** 10.1093/bioinformatics/btac717

**Published:** 2022-11-07

**Authors:** Jimeng Lei, Zongheng Cai, Xinyi He, Wanting Zheng, Jianxiao Liu

**Affiliations:** National Key Laboratory of Crop Genetic Improvement, Huazhong Agricultural University, Wuhan 430070, China; Key Laboratory of Smart Farming for Agricultural Animals, Huazhong Agricultural University, Wuhan 430070, China; College of Informatics, Huazhong Agricultural University, Wuhan 430070, China; National Key Laboratory of Crop Genetic Improvement, Huazhong Agricultural University, Wuhan 430070, China; Key Laboratory of Smart Farming for Agricultural Animals, Huazhong Agricultural University, Wuhan 430070, China; College of Informatics, Huazhong Agricultural University, Wuhan 430070, China; College of Informatics, Huazhong Agricultural University, Wuhan 430070, China; College of Informatics, Huazhong Agricultural University, Wuhan 430070, China; National Key Laboratory of Crop Genetic Improvement, Huazhong Agricultural University, Wuhan 430070, China; Key Laboratory of Smart Farming for Agricultural Animals, Huazhong Agricultural University, Wuhan 430070, China; College of Informatics, Huazhong Agricultural University, Wuhan 430070, China; Hubei Key Laboratory of Agricultural Bioinformatics, Huazhong Agricultural University, Wuhan 430070, China

## Abstract

**Motivation:**

The question of how to construct gene regulatory networks has long been a focus of biological research. Mutual information can be used to measure nonlinear relationships, and it has been widely used in the construction of gene regulatory networks. However, this method cannot measure indirect regulatory relationships under the influence of multiple genes, which reduces the accuracy of inferring gene regulatory networks.

**Approach:**

This work proposes a method for constructing gene regulatory networks based on mixed entropy optimizing context-related likelihood mutual information (*MEOMI*). First, two entropy estimators were combined to calculate the mutual information between genes. Then, distribution optimization was performed using a context-related likelihood algorithm to eliminate some indirect regulatory relationships and obtain the initial gene regulatory network. To obtain the complex interaction between genes and eliminate redundant edges in the network, the initial gene regulatory network was further optimized by calculating the conditional mutual inclusive information (*CMI2*) between gene pairs under the influence of multiple genes. The network was iteratively updated to reduce the impact of mutual information on the overestimation of the direct regulatory intensity.

**Results:**

The experimental results show that the *MEOMI* method performed better than several other kinds of gene network construction methods on DREAM challenge simulated datasets (DREAM3 and DREAM5), three real *Escherichia coli* datasets (*E.coli* SOS pathway network, *E.coli* SOS DNA repair network and *E.coli* community network) and two human datasets.

**Availability and implementation:**

Source code and dataset are available at https://github.com/Dalei-Dalei/MEOMI/ and http://122.205.95.139/MEOMI/.

**Supplementary information:**

Supplementary data are available at *Bioinformatics* online.

## 1 Introduction

Inferring gene regulatory networks (*GRNs*), also known as reverse engineering, is a critical problem in computational biology. The gene regulatory network mainly refers to the interaction network formed by gene regulation in cells or within a specific genome. It is critical to accurately infer *GRNs* to understand biological activity and discover the new pathways. With the rapid development of next-generation sequencing technologies, it provides previously unexplored opportunities for research into gene regulatory relationships. How to construct a gene regulatory network at the biomolecular level is a complicated problem in biological research (Novere, 2015).

Building an appropriate mathematical or machine learning model using gene expression data and reverse engineering is a commonly used method for inferring gene regulatory networks. To date, numerous computational approaches for inferring gene regulatory networks have been developed. The computational approaches can be roughly categorized into model-based methods and information theory-based methods (Liu *et al.*, 2016). Model-based approaches can be approximately classified into three categories: statistical methods, machine learning methods and probabilistic graphical model methods. Statistical methods were the earliest methods used to construct gene regulatory networks. The representative methods include analysis of variance (*ANOVA*) (Küffner *et al.*, 2012), partial least squares regression (Li *et al.*, 2012), nonlinear autoregressive models, distance correlation (Guo *et al.*, 2014), linear and probabilistic relations prediction and linear mapping approximation (Cao and Grima, 2018). *GENIE3* is a classical and widely used machine learning method for constructing gene networks. It uses random forests to predict the expression pattern of target genes based on the expression patterns of other genes (Huynh-Thu *et al.*, 2010). Some research work has been performed to improve *GENIE3*, including *BTNET* (Park *et al.*, 2018), *BiXGBoost* (Zheng *et al.*, 2019), *GRNBoost2* and *Arboreto* (Moerman *et al.*, 2019). In recent years, deep learning methods have become increasingly popular in the construction of gene regulatory networks. *GRGNN* uses a graph neural network to reconstruct *GRN* utilizing gene expression data (Wang *et al.*, 2020). *GripDL* uses a deep learning method to predict *GRN* using a gene expression image dataset (Yang *et al.*, 2019). In terms of probabilistic graphical model methods, Schäfer and Strimmer (2005) used graphical Gaussian models (*GGMs*) to infer gene regulatory networks on the basis of a small number of samples. *LDGM* uses a differential graphical model to identify gene regulatory networks (Tian *et al.*, 2016). Liu *et al.* (2016) inferred the *GRN* from gene expression data by using a network decomposition strategy and a false-positive edge elimination scheme. In addition, Bayesian networks and dynamic Bayesian networks (*DBNs*) have been used extensively in research to identify gene regulatory networks based on time-course microarray data (Liu *et al.*, 2016).

Mutual information is the most widely used information theory method for constructing gene regulatory networks. This method requires no prior knowledge, and it can process a large number of genes, and it has a better learning effect on nonlinear relationships between genes. The relevance network was the earliest method that used mutual information to infer gene regulatory networks (Butte and Kohane, 2000). *ARACNE* uses data processing inequality based on mutual information to filter out the indirect edges, allowing it to efficiently handle three-gene loops (Margolin *et al.*, 2006). Some subsequent studies have improved *ARACNE*, including *TimeDelay*-*ARACNE*, *ARACNe*-*AP* (Lachmann *et al.*, 2016), etc. The context likelihood of relatedness (*CLR*) algorithm considers the cumulative distribution of mutual information between two genes and other genes when calculating the correlation between the two genes (Daub *et al.*, 2004). Researchers later improved the *CLR* algorithm, including *SA-CLR*, *MixedCLR* and *DeGNServer*. *MRNET* (Meyer *et al.*, 2007), *MRNETB* and *RRMRNET* select characteristic genes by finding the genes in a gene set that have the highest correlation with the final result (max-relevance), but the lowest correlation between genes (min-redundancy). *NARROMI* improves the *GRN* inference accuracy by combining ordinary differential equation-based recursive optimization (*RO*) and information theory-based mutual information (Zhang *et al.*, 2013). In recent years, some of the studies infer *GRNs* through combining path consistency algorithm and conditional mutual information, including *PCA*-*CMI* (Zhang *et al.*, 2012), *IPCA*-*CMI* (Aghdam *et al.*, 2014) and *CMI2NI* (Zhang *et al.*, 2015). Among them, Zhang *et al.* (2015) developed conditional mutual inclusion information (*CMI2*) to describe the regulatory relationship between genes, which involves calculating the information loss of a hypothetical distribution in the presence or absence of edges. Later, some studies have solved order dependency issue in the path consistency algorithm for inferring *GRN*, including *SORDER* (Aghdam *et al.*, 2015) and *OIPCQ* (Mahmoodi *et al.*, 2021).

However, mutual information overestimates the direct regulatory relationship between genes to a certain extent. That is to say, mutual information cannot distinguish indirect and direct gene regulatory relationships, leading to a higher false-positive rate. Although conditional mutual information (*CMI*) can distinguish direct and indirect regulatory relationships, it often underestimates the intensity of gene regulation (Janzing *et al.*, 2013; Marbach *et al.*, 2012; Reshef *et al.*, 2011; Runge *et al.*, 2012). To solve the above problems, this work proposes a gene regulatory network construction method based on mixed entropy optimizing context-related likelihood mutual information (*MEOMI*).

1. The James–Stein entropy estimation without prior distributions and Bayes entropy estimation based on Dirichlet prior distributions were combined to calculate the mutual information between genes. Then, it performs the distribution optimization of mutual information matrix using the CLR algorithm, thus obtaining the initial gene regulatory network with relatively high accuracy.
2. The conditional mutual inclusive information between genes under the influence of multiple genes was calculated. The path consistency algorithm was used to traverse the entire gene regulatory network. The redundant edges were deleted gradually by dynamically setting the thresholds, resulting in a more accurate gene regulatory network.
3. *MEOMI* was compared with eight kinds of gene network construction methods (*GENIE3*, *CLR*, *ARACNE*, *MRNET*, *CMI2NI*, *NARROMI*, *BiXGBoost* and *PIDC*) using the commonly used DREAM challenge dataset, three real *Escherichia coli* datasets and two human datasets. The experimental results show that the *PPV*, *ACC*, *MCC*, *F1-score*, *AUPR* and *AUC* of the *MEOMI* method were better than those of the other methods in the different datasets.

## 2 Materials and methods

Entropy estimation is also a popular research topic in the field of gene network construction (Daub *et al.*, 2004). As shown in Supplementary Section S3, the mutual information depends on the entropy calculation. To enhance the accuracy of the entropy calculation, this work uses a hybrid entropy estimation method to calculate the entropy.

### 2.1 Mixed entropy calculation

#### 2.1.1 James–Stein shrinkage estimation without a prior distribution

James–Stein shrinkage estimation is suitable for gene network inference calculation for high-dimensional datasets. That is to say, James–Stein shrinkage estimation has a better calculation effect for datasets with a small number of samples. James–Stein contraction estimation ensures that the mean square error is minimized by adding weight to two different models, as shown in Equation (3). The two models refer to a high-dimensional model with a low deviation and a high variance, and a low-dimensional model with a low deviation and a high variance.

Maximum likelihood estimation (*ML*) is a classical entropy estimation method. The maximum likelihood entropy ${\hat{H}}^{ML}(X)$ is calculated using Equation (1), in which ${\hat{\theta }}^{ML}(x_{k})$ denotes the probability parameter.

(1) $${\hat{H}}^{ML}(X)=-\sum_{k\in X}{\hat{\theta }}^{ML}(x_{k})\;\text{log}\;{\hat{\theta }}^{ML}(x_{k})$$

The polynomial distribution probability model of the correlation between the count of each bin $n(x_{i})$ and the corresponding probability parameter $\theta (x_{i})$ is shown in Equation (2) (Hausser, 2006).

(2) $$\mathrm{Prob}(n(x_{1}),\ldots ,n(x_{K});\theta (x_{1}),\ldots \theta (x_{K}))=\frac{n!}{\prod_{i=1}^{K}n(x_{i})!}\prod_{i=1}^{K}\theta (x_{i})$$

*ML* estimation can be performed for parameter $\theta (x_{i})$ by taking the maximum value of the likelihood function on the right side of Equation (2). Then, we can obtain the maximum likelihood estimation value of $\theta (x_{i})$, which is denoted as ${\hat{\theta }}^{ML}(x_{i})=n(x_{i})/n$. It can also be proven that the variance $\mathrm{Var}({\hat{\theta }}^{ML}(x_{i}))=({\hat{\theta }}^{ML}(x_{i})(1-{\hat{\theta }}^{ML}(x_{i})))/(n-1)$, the deviation $\mathrm{Bias}({\hat{\theta }}^{ML}(x_{i}))=0$, and $E({\hat{\theta }}^{ML}(x_{i}))=\theta (x_{i})$. The *ML* estimation is an unbiased model with high variance.

The entropy calculation using maximum likelihood estimation is based on asymptotic theory. When the sample size is small, the count in the bin is more likely to be 0. Then, the probability under the maximum likelihood estimation will also be 0, affecting the calculation accuracy. This work reduces the *ML* estimation value to *t_k_* based on James–Stein estimation, and calculates the shrinkage strength *λ* by minimizing the mean square error (*MSE*). This can help to improve the calculation accuracy and achieve better performance than other single estimators (Hausser, 2006), as shown in Equation (3).

(3) $${\hat{\theta }}^{JS}(x_{k})=\lambda t_{k}+(1-\lambda ){\hat{\theta }}^{ML}(x_{k})$$

In Equation (3), λ is the shrinkage strength, and its value is between 0 and 1. *t_k_* is the corresponding shrinkage target. In general, $t_{k}=1/K$, and it corresponds to the uniform distribution of the maximum entropy target. Moreover, the shrinking target *t* has no variance but has a higher deviation (Hausser, 2006). The first step in determining the optimal contraction strength *λ* is to choose a suitable loss function. In this work, we used the square error as the loss function. The second step is to minimize the risk function *R*(*λ*), in this work, we used the mean square error, as shown in Equation (4).

(4) $$R(\lambda )=E(L(\lambda ))=E{\left(\sum_{k=1}^{p}\left(\hat{\theta }(x_{k}\right)-\theta (x_{k})\right)}^{2})$$

We can obtain the shrinkage strength *λ* that minimizes the *MSE*, as shown in Equation (5).

(5) $$\begin{array}{c} \hat{\lambda }=\frac{\sum_{k=1}^{p}\mathrm{Var}({\hat{\theta }}^{ML}(x_{k}))-\mathrm{Cov}({\hat{\theta }}^{ML}(x_{k}),t_{k})}{\sum_{k=1}^{p}E[{\left({\hat{\theta }}^{ML}(x_{k})-t_{k}\right)}^{2}]}\\ \;+\frac{\mathrm{Bias}({\hat{\theta }}^{ML}(x_{k}))E({\hat{\theta }}^{ML}(x_{k})-t_{k})}{\sum_{k=1}^{p}E[{\left({\hat{\theta }}^{ML}(x_{k})-t_{k}\right)}^{2}]} \end{array}$$

Given that $\mathrm{Var}({\hat{\theta }}^{ML}(x_{k}))=({\hat{\theta }}^{ML}(x_{k})(1-{\hat{\theta }}^{ML}(x_{k})))/(n-1)$ and $\mathrm{Bias}({\hat{\theta }}^{ML}(x_{k}))=0$, we can obtain Equation (6).

(6) $$\hat{\lambda }=\frac{\sum_{k=1}^{p}\mathrm{Var}({\hat{\theta }}^{ML}(x_{k}))}{\sum_{k=1}^{p}{\left({\hat{\theta }}^{ML}(x_{k})-t_{k}\right)}^{2}}=\frac{1-\sum_{k=1}^{p}{\hat{\theta }}^{ML}{\left(x_{k}\right)}^{2}}{(n-1)\sum_{k=1}^{p}{\left({\hat{\theta }}^{ML}(x_{k})-t_{k}\right)}^{2}}$$

To avoid excessive shrinkage, we set ${\hat{\theta }}^{JS}(x_{k})=1/K$ when $\hat{\lambda }\geq 1$. When a negative contraction occurs, we set $\hat{\lambda }=0$, and $0\leq \hat{\lambda }\leq 1$ can be satisfied.

#### 2.1.2 Bayesian polynomial ratio estimation based on a Dirichlet prior distribution

The Dirichlet distribution is also called as the multivariate beta distribution and can be used to estimate the entropy of discrete data. It is a high-dimensional generalization of the beta distribution. The density form of a Dirichlet prior distribution with parameters *a*_1_ to *a_K_* and dimension *K* is shown in Equation (7).

(7) $$f(\theta_{1},\ldots ,\theta_{K};a_{1},\ldots ,a_{K})=\frac{\Gamma (\sum_{i=1}^{K}a_{i})}{\prod_{i=1}^{K}\Gamma (a_{i})}\prod_{i=1}^{K}\theta_{i}^{a_{i}-1}\delta \left(1-\sum_{i=1}^{K}\theta_{i}\right)$$

In Equation (7), *a_i_* is the prior parameter of event *θ_i_*, and *Γ*(·) is the gamma function. The definition of *δ* is shown in Equation (8).

(8) $$\delta (x)=\left\{ \begin{array}{c} 1\;if\;\;x=0\\ \;0\;\mathrm{otherwise} \end{array}\right.$$

If there is no prior knowledge, all priori parameters *a_i_* have the same default value. This means that all events have the same probability of occurring under unknown conditions, thus, $a_{1}=a_{2}=\cdots =a_{K}=a$, and $m=\sum_{i=1}^{K}a_{i}$. The Bayesian estimation based on the Dirichlet prior distribution is shown in Equation (9).

(9) $${\hat{\theta }}_{i}^{\mathrm{Dir}}=\frac{n(x_{i})+a_{i}}{m+n}$$

In general, we directly estimated the entropy value based on the estimated probability *θ* in Equation (9). However, the accuracy of the entropy estimation obtained using this method is unknown. Therefore, we directly estimated the entropy from the existing data. Then, the entropy $H(\hat{\theta })$ calculated based on the estimated probability *θ* is not equivalent to the estimated entropy $\hat{H}(\chi )$ calculated directly based on the index vector. To obtain the estimated entropy quickly and effectively, we first obtained the equation of the *β*-th moment of the probability distribution, as shown in Equation (10).

(10) $$E(\theta_{j}^{\beta })=\frac{\Gamma (n(x_{j})+\beta +a)\Gamma (n+Ka)}{\Gamma (n(x_{j})+a)\Gamma (n+Ka+\beta )}$$

In Equation (10), *β* denotes the moment, *n*(*x_j_*) represents the count of each bin, and *K* is the number of bins with counts greater than 0. Based on $\partial (\theta_{j}^{\beta })/\partial \beta {|}_{\beta =1}=\theta_{j}\ln\theta_{j}$ and Dirichlet prior distribution, the entropy estimator ${\hat{H}}^{\mathrm{Dir}}(X)$ can be calculated through Equation (11).

(11) $$\begin{array}{c} {\hat{H}}^{\mathrm{Dir}}(X)=\frac{1}{m+Ka}\sum_{k\in X}\left(n(x_{k})+a\right)\cdot \\ \sum_{k\in X}\left(\psi (m+Kn+1)-\psi (n(x_{k})+a+1)\right) \end{array}$$

In this equation, ψ(z)=d ln Γ(z)/dz is the psi function. We can see that the calculation paradigm depends on the count vector and the priori parameters of the discrete data. The calculation process is simple and easy to implement. The most critical step is determining the parameter *a* of the prior distribution, which is equivalent to adding a pseudo count to *K* cells. To obtain the value of the prior parameter *a*, we considered the relationship between the prior parameter and the contraction strength *λ*.

By setting λ=m/(n+m) and ${\hat{\theta }}_{i}^{\mathrm{Dir}}={\hat{\theta }}_{i}^{JS}$, we can obtain the corresponding relationship between the contraction intensity λ estimated by James–Stein contraction estimation and the Dirichlet prior parameter *a*, as shown in Equation (12).

(12) $$a=\frac{n}{K}\left(\frac{1-\lambda }{\lambda }\right)$$

There are numerous options for *a_i_* in entropy estimation. If $a_{i}=0$, the entropy estimation is a classical *ML* estimation without prior knowledge (Meyer *et al.*, 1997). If $a_{i}=\sqrt{n}/K$, the entropy estimation is a maximum-minimum likelihood estimation. If $a_{i}=\frac{1}{2},a_{i}=1,a_{i}=\frac{1}{K},\mathrm{for}\;\;i=1,\ldots ,K$, these values are reasonable estimates under an unknown prior (Chiu and Ted, 1991; Jeffreys, 1946; SchüRmann and Grassberger, 1996).

### 2.2 Mutual information matrix optimization

In this section, we further processed the obtained mutual information matrix *MIM* in order to obtain a more accurate initial gene regulatory network. By considering the cumulative distribution of the mutual information between genes, we eliminated some indirect regulatory relationships between genes in the network (Faith *et al.*, 2007). The *Z* score *Z_i_* of gene *G_i_* was calculated through Equation (13).

(13) $$Z_{i}=\max\left(0,\frac{I(X_{i};X_{j})-\mu_{i}}{\sigma_{i}}\right)$$

In the equation, $I(X_{i};X_{j})$ represents the mutual information of the gene pair *G_j_* and *G_i_*. The random variables corresponding to any two genes *G_i_* and *G_j_* were denoted as *X_i_* and *X_j_*, respectively. The mutual information of all *G_j_* associated with *G_i_* in *MIM* were formed into an empirical distribution, and *Z_i_* was obtained after standardizing the distribution. In Equation (13), *μ_i_* and *σ_i_* represent the mean and standard deviation, respectively. To obtain the *Z_i_* of a single gene, we calculated the likelihood mutual information scores of gene *G_i_* and *G_j_*, as shown in Equation (14).

(14) $$Z_{ij}=\sqrt{Z_{i}^{2}+Z_{j}^{2}}$$

Finally, we obtained a score *Z_ij_* related to the empirical distribution of the mutual information based on the mutual information of each pair of genes. The matrix of *Z_ij_* scores replaced *MIM* as the initial weight matrix of the gene regulatory network in the next step to remove the redundant edges.

### 2.3 Removing the redundant edges

**Fig. 1.**
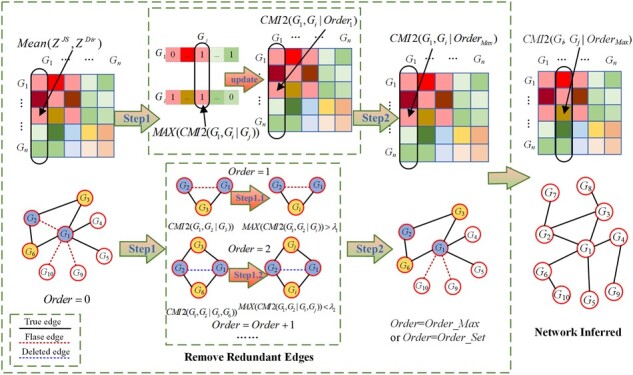
The process of removing the redundant edges. For each edge of gene pairs (eg. *G*_1_ and *G*_2_) in the initial gene regulatory network, it finds all adjacent genes *G_i_* that have edges with *G*_1_ and *G*_2_ firstly. It calculates the conditional interaction information *CMI2*(*G*_1_, *G*_2_ | *G_i_*) iteratively. The redundant edges are deleted gradually through setting thresholds dynamically, and it obtains more accurate gene regulatory network step by step

The mutual information method is incapable of effectively handling indirect regulatory relationships between genes, leading to a high false-positive rate in the constructed gene regulatory network. We used the conditional mutual inclusive information calculation method to gradually remove redundant edges (Zhang *et al.*, 2015). The conditional mutual inclusive information of a pair of genes under the influence of multiple related genes is calculated by multi-order traversal algorithm. The redundant edges were gradually removed, resulting in a more accurate gene regulatory network. The specific implementation process is shown in Figure 1.

As shown in Figure 1, for gene *G*_1_, there was an edge between *G*_1_ and *G*_2_ in the initial gene regulatory network. Then, the algorithm searched for genes *G_i_* with edges with both *G*_1_ and *G*_2_. Suppose the total number of genes *G_i_* that satisfied the above conditions was *Order_Max*, that is, the maximum number of genes that interact with *G*_1_ and *G*_2_ is *Order_Max*. If *Order *=* *0, the initial gene regulatory network was obtained based on the initial threshold *λ*_0_, and *Order* was gradually increased to incrementally update the gene regulatory network.

Starting with *Order *=* *1, the algorithm calculated the conditional interaction information *CMI2*(*G*_1_, *G*_2_ | *G_i_*) for all *G_i_*, and returned the maximum value as *MAX*(*CMI2*(*G*_1_, *G*_2_ | *G_i_*)). If *MAX*(*CMI2*(*G*_1_, *G*_2_ | *G_i_*)) was less than the threshold *λ*_1_, the algorithm deleted the edge between *G*_1_ and *G*_2_, and set the value of the gene adjacency matrix *G*_1_(1, 2) to 0. If *MAX*(*CMI2*(*G*_1_, *G*_2_ | *G_i_*)) was greater than the threshold *λ*_1_, the algorithm increased the value of *Order*. It calculated *CMI2*(*G*_1_, *G*_2_ | *G_i_*) for *Order* genes and compared the results with the threshold *λ_i_* in turn according to the above steps until *Order=Order_Max* or *Order* reached the maximum value of *Order_Set*. Then, the algorithm traversed all the genes according to the above steps. The incorrect edges were deleted gradually, resulting in a more accurate gene regulatory network.

The threshold has a great impact on the experimental results. In this work, we use the dynamic setting threshold approach to screen the redundant regulatory relationships between genes. The larger the threshold, the stricter the control over the gene regulatory relationships. Given the influence of multiple indirect regulatory relationships on the direct regulatory relationship between genes, when calculating *CMI2*(*G_i_*, *G_j_* | *G_k_*) for each order, we considered both the initial threshold *α* and the combination number *m* of *Order* genes extracted from *Order_Max*. Obviously, *m* depends on the value of *Order* and the number of all associated genes *Order_Max*. The larger the value of *m*, the more indirect regulations are affected, thereby increasing the corresponding threshold.

In Zhang *et al.* (2015), for random variables *X* and *Y* given *Z*, CMI2(X;Y|Z) was calculated using Equation (15).

(15) $$\begin{array}{c} CMI2(X;Y|Z)=\frac{1}{2}(D_{KL}(\theta (X,Y,Z)||\theta_{X\to Y}(X,Y,Z))\\ \;\;\;\;\;\;\;+D_{KL}(\theta (X,Y,Z)||\theta_{Y\to X}(X,Y,Z))) \end{array}$$

In the equation, $D_{KL}(\theta (X,Y,Z)||\theta_{X\to Y}(X,Y,Z))$ is the KL divergence from θ(X,Y,Z) to $\theta_{X\to Y}(x,y,z)$, which is used to measure the distance between the two distributions. θ(X,Y,Z) represents the joint probability among *X*, *Y* and *Z*. $\theta_{X\to Y}(x,y,z)$ is the interference probability from *X* to *Y*. In addition, the causal variable intensity between *X* and *Y* is shown in Equation (16).

(16) $$C_{X\to Y}(X;Y|Z)=D_{KL}(\theta (X,Y,Z)||\theta_{X\to Y}(X,Y,Z))$$

$C_{X\to Y}(X;Y|Z)$ is asymmetric, and CMI2(X;Y|Z) is the average of $C_{X\to Y}(X;Y|Z)$ and $C_{Y\to X}(Y;X|Z)$. After expanding Equation (16), we can obtain Equation (17).

(17) $$\begin{array}{c} D_{KL}(\theta (X,Y,Z)||\theta_{Y\to X}(X,Y,Z))\\ =I(X;Y|Z)+D_{KL}(\theta (X|Z)||\theta_{Y\to X}(X|Z)) \end{array}$$

By combining Equation (18) and Equation (15), we can obtain CMI2(X;Y|Z), as shown in Equation (18).

(18) $$\begin{array}{c} CMI2(X;Y|Z)=I(X;Y|Z)+\frac{1}{2}D_{KL}(\theta (Y|Z)||\theta_{X\to Y}(Y|Z))\\ +\frac{1}{2}D_{KL}(\theta (X|Z)||\theta_{Y\to X}(X|Z)) \end{array}$$

$\theta_{X\to Y}(Y|Z)$ can be calculated by Equation (19).

(19) $$\theta_{X\to Y}(y|Z)=\sum_{x}\theta (y|x,z)\theta (x)=\sum_{x}\theta (y|x,z)\theta (x|z)=\theta (y|z)$$

In order to estimate the unknown density function in Equation (19), we used the Gaussian kernel density estimation method to calculate the corresponding density function. When using the Gaussian kernel density method to estimate the probability density function, we applied the broad assumption that the gene expression data were subject to a multivariate Gaussian distribution to perform calculations (Zhang *et al.*, 2015). This can aid in achieving a more efficient estimation of CMI2(X;Y|Z), and the concrete calculation process can be found in Zhang *et al.* (2015).

## 3 Results

We compared the *TP*, *FP*, *TN*, *FN*, *TPR*, *PPV*, *F1*-*score*, *FPR*, *ACC*, *MCC*, *AUPR* and *AUC* of the different methods in all the datasets. Detailed information can be found in Supplementary Section S4. We compare *MEOMI* with nine kinds of different methods (*GENIE3-RF-sqrt* (Huynh-Thu *et al.*, 2010), *GENIE3-RF-all* (Huynh-Thu *et al.*, 2010), *CLR* (Faith *et al.*, 2007), *ARACNE* (Margolin *et al.*, 2006), *MRNET* (Meyer *et al.*, 2007), *CMI2NI* (Zhang *et al.*, 2015), *NARROMI* (Zhang *et al.*, 2013), *BiXGBoost* (Zheng *et al.*, 2019) and *PIDC* (Chan *et al.*, 2017)).

### 3.1 Datasets

**DREAM Challenge dataset**. The DREAM challenge dataset (DREAM3 and DREAM5) includes a series of gene expression data with noise and reference networks selected from real source networks (Schaffter *et al.*, 2011). The standard network in this dataset is the experimentally verified yeast and *E.coli* regulatory network. There were 50/100 (denoted as Size50 and Size100, respectively) genes and 50/100 samples in DREAM3, and they included 5 groups of data: Ecoli1, Ecoli2, Ecoli3, Yeast1 and Yeast2. Each group included two data types: heterozygous and null-mutants. For each data type, we used 5 datasets to perform experimental comparisons. Detailed information of the Size50/100 datasets are shown in Table 1.

**Table 1.** Size50/100 dataset information in DREAM3

ID	Dataset information	Data	Dataset information
Data1	InSilicoSize50/100-Ecoli1-heterozygous	Data6	InSilicoSize50/100-Yeast1-null-mutants
Data2	InSilicoSize50/100-Ecoli1-null-mutants	Data7	InSilicoSize50/100-Yeast2-heterozygous
Data3	InSilicoSize50/100-Ecoli2-heterozygous	Data8	InSilicoSize50/100-Yeast2-null-mutants
Data4	InSilicoSize50/100-Ecoli2-null-mutants	Data9	InSilicoSize50/100-Yeast3-heterozygous
Data5	InSilicoSize50/100-Yeast1-heterozygous	Data10	InSilicoSize50/100-Yeast3-null-mutants

The DREAM5 dataset includes 1643 genes (marked as Size1643), 805 samples and 195 transcription factors. Considering that only transcription factors are involved in standard networks, we used only the top regulatory relationships related to the 195 transcription factors to perform experimental comparisons.

**Fig. 2.**
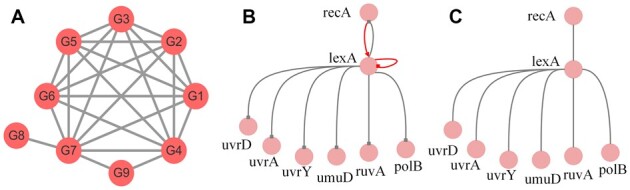
The standard network of two real *E.coli* datasets. (**A**) The standard network of *E.coli* SOS pathway dataset. (**B**) The standard network of *E.coli* SOS DNA repair dataset. (**C**) Without considering the direction and self-loop, the standard network used in this work about the *E.coli* SOS DNA repair dataset

***Escherichia coli* dataset**. The *E.coli* SOS network is a well-studied biological network that is widely used to assess *GRN* inference techniques. The first *E.coli* dataset is denoted as *E.coli* SOS DNA pathway dataset, which includes nine genes and nine samples. There are 9 genes (*G1*–*G9*) and 24 edges in the standard network, in which are the principle mediators, known regulatory genes and sigma factor genes involved in the SOS response (Guo *et al.*, 2014; Zhang *et al.*, 2015). The standard network of the *E.coli* SOS pathway dataset is shown in Figure 2A.

The second *E.coli* SOS dataset included eight genes (*uvrD*, *lexA*, *umuD*, *recA*, *uvrA*, *uvrY*, *ruvA* and *polB*) that are significantly involved in the DNA repair process. The original standard network of the *E.coli* SOS DNA repair dataset is shown in Figure 2B. The standard network used in this work without considering direction and self-loop is shown in Figure 2C. This dataset was generated by setting a certain time step in the experiment (the step length was 50), and sampling the gene interval under different ultraviolet light intensities. There were four experiments in total, and the preprocessing of the dataset retained the first time point (0) (Larvie *et al.*, 2016; Ronen *et al.*, 2002).

The third *E.coli* dataset was the *E.coli* community network (Faith *et al.*, 2008), the expression profile dataset used was retrieved from many microbial microarray (M3D) databases (version 4, build 6). The *E.coli* community network dataset contains 907 chip measurements of 4297 genes collected from different experiments at steady state levels (Huynh-Thu *et al.*, 2010). Compared with other datasets, the M3D database provides fewer datasets but with the same data preprocessing, thus this database is suitable for the construction of a gene regulatory network (Küffner *et al.*, 2012). Detailed results of the *E.coli* community network dataset are shown in Supplementary Table S10.

The *E.coli* SOS DNA repair network involves 4 datasets, with each dataset having 8 genes and 50 samples. The information of the three real *E.coli* datasets is shown in Table 2.

**Table 2.** The information of three real *E.coli* datasets

ID	Dataset	ID	Dataset
EData1	*E.coli* SOS pathway network	EData2-3	*E.coli* SOS DNA repair network-3
EData2-1	*E.coli* SOS DNA repair network-1	EData2-4	*E.coli* SOS DNA repair network-4
EData2-2	*E.coli* SOS DNA repair network-2	EData3	*E.coli* community network

**Human gonadal sex determination (GSD) and mature hepatocytes (hHEP) datasets.** The GSD dataset included 19 genes, 79 edges and 2000 samples (Pratapa *et al.*, 2020). The hHEP dataset includes 11 515 genes and 425 samples. We selected the top 100, 200, 500 most variable genes to do experimental comparison, and there are 9, 17, 33 TF genes, respectively in each datasets. Detailed results of human dataset are shown in Supplementary Section S7.

### 3.2 Experimental comparisons on the DREAM challenge dataset

#### 3.2.1 Experiment results of the Size50 datasets

**Fig. 3.**
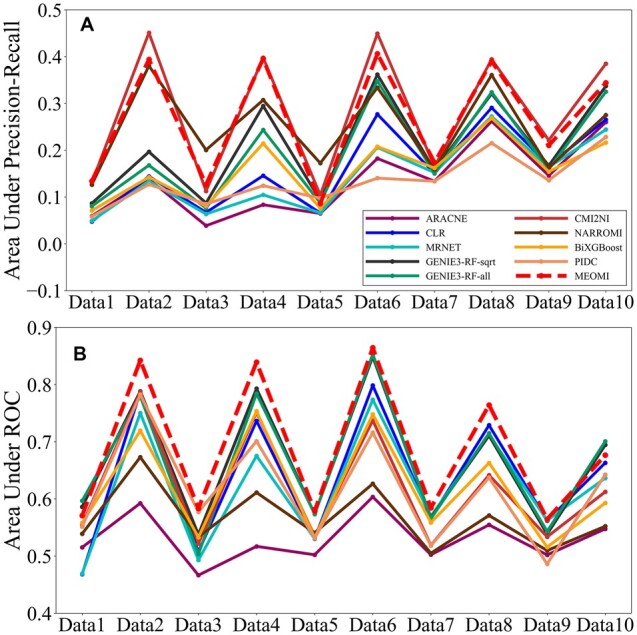
The AUPR and AUROC comparison results on the DREAM3 Size50 datasets

The *MEOMI* method has three parameters. The first parameter is *bins*, which denotes the dispersion of the dataset. Based on our preliminary experimental results, the parameter *bins* was generally set to 5. The second parameter *order* is the number of polygenes that control the direct regulation of gene pairs. The algorithm will terminate when *order* reaches a certain threshold. The value of *order* has a great impact on the running time of the algorithm, and setting a larger *order* leads to a low efficiency of *MEOMI*. In general, we set *order *=* *2 or *order *=* *3. The third parameter *λ* refers to the initial threshold *λ*_0_ used to screen the edges in the gene regulatory network (step 40 in Supplementary Algorithm S1). Based on our preliminary experimental results, the range of *λ* was generally set to 0.000001–0.01. In addition, we found that the value of *λ* was mainly related to the number of genes in the dataset. The greater the number of genes, the larger the value of *λ*. For the Size50 dataset, we set *λ *= 0.000001 in the *MEOMI* algorithm. Figure 3 shows the *AUPR* and *AUC* comparison results for the nine different methods.

**Fig. 4.**
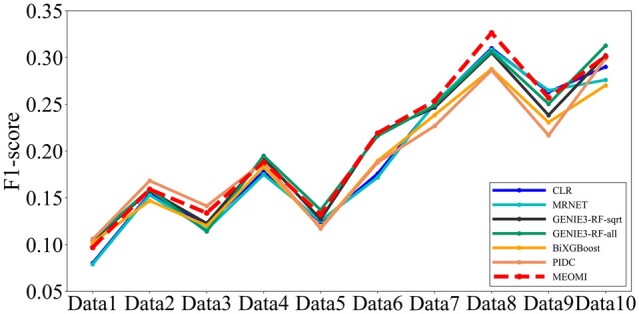
The F1-score comparison results of six kinds of methods on Size50

In Figure 3B, the *AUC* of the *MEOMI* method is essentially higher than those of the other 9 methods in the 10 datasets. The *AUC* of the *MEOMI* method was slightly lower than the *AUC* of *GENIE3* method in Data1 and Data10. However, the difference between the two methods was very small. Figure 3A shows that the *AUPR* of the *CMI2NI* and *MEOMI* method is quite good and are overall higher than those of the other eight approaches. Except for a few datasets, the *AUPR* of the *MEOMI* method is similar to that of the *CMI2NI* method, and both are better than the *AUPR* of the other methods. Although *CMI2NI* performed better than *MEOMI* on *AUPR*, its *TPR* on the 10 datasets were all lower than the *TPR* of *MEOMI*. The detailed results can be seen in Supplementary Section S5.1. The *TPR* of *CMI2NI*, *NARROMI* and *ARACNE* is lower than other seven kinds of methods apparently. It means that these three kinds of approach cannot obtain more number of correct edges, and we only give the comparison results of *GENIE3-RF-sqrt*, *GENIE3-RF-all*, *CLR*, *MRNET*, *BiXGBoost* and *PIDC*. Figure 4 elaborates the *F1-score* comparison result of Size50 dataset about the above 7 kinds of methods.

It can be seen that the *F1-score* of *MEOMI* is superior to *GENIE3-RF-sqrt*, *GENIE3-RF-all*, *CLR*, *MRNET*, *BiXGBoost* and *PIDC* on most of the data while ensuring that there are more number of correct edges in the network.

#### 3.2.2 Experiment results of the Size100 datasets

**Fig. 5.**
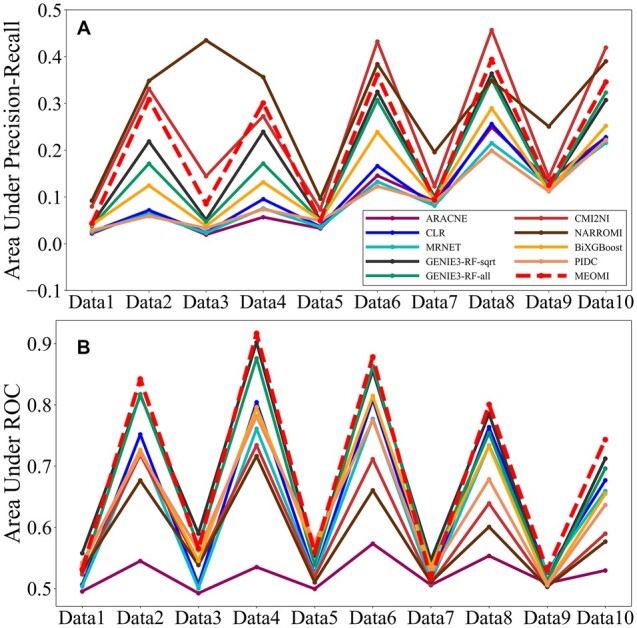
The AUPR and AUROC comparison results on the DREAM3 Size100 datasets

For the Size100 dataset, *λ* was also set to 0.000001 in *MEOMI*. Figure 5 shows the *AUPR* and *AUC* comparison results for the 10 different methods.

**Fig. 6.**
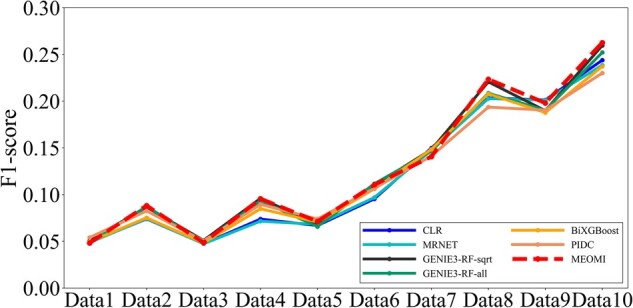
The F1-score comparison results of six kinds of methods on Size100

Figure 5B shows that the *AUC* of the *MEOMI* method was better than those of the other methods on the different datasets. The overall result trend was roughly the same as that of *GENIE3-RF-sqrt*, and the performance on some heterozygous data was slightly lower than that of *GENIE3-RF-sqrt*. The *AUC* of all methods was less than 0.6 on the 5 heterozygous datasets, indicating that the experimental data were not very characteristic. In addition, we can see that the result of the *GENIE3-RF-sqrt* method was better than that of the *GENIE3-RF-all* method. This implies that selecting features randomly has a greater impact on the experimental results of the heterozygous datasets. In Figure 5A, the *AUPR* of *MEOMI* was slightly lower than *CMI2NI* and *NARROMI* on some datasets. However, the *AUPR* of *MEOMI* was better than the other seven kinds of methods. Although *CMI2NI* and *NARROMI* performed better than *MEOMI* in term of *AUPR*, the corresponding *TPR* on the 10 datasets was lower than *MEOMI*. The detailed results can be seen in Supplementary Section S5.1. Similarly, Figure 6 elaborates the *F1-score* comparison results of Size100 dataset about seven kinds of methods.

Through Figure 6, we can see that *MEOMI* performing better than *GENIE3-RF-sqrt*, *GENIE3-RF-all*, *CLR*, *MRNET*, *BiXGBoost* and *PIDC* on the whole.

#### 3.2.3 Experimental results of the DREAM5 dataset

**Fig. 7.**
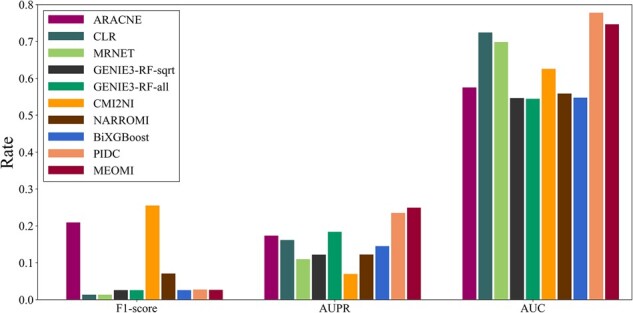
F1-score, AUPR and AUC comparison of the nine methods on DREAM5 dataset

The experimental results of the DREAM5 dataset are shown in Figure 7. Detailed information can be seen in Supplementary Section S5.4. As described above, the parameter *λ* was mainly related to the number of genes in the dataset. The greater the number of genes, the greater the value of *λ*. For the DREAM5 dataset, which included more genes than the DREAM3 dataset, *λ* was set to 0.001.

Figure 7 shows that the *F1*-*score* of *MEOMI* was lower than the *F1*-*score* of *NARROMI*, *CMI2NI* and *ARACNE* on the DREAM5 dataset, but it was higher than the *F1*-*score* of the other methods. *NARROMI*, *CMI2NI* and *ARACNE* all used mutual information to perform calculation and construct the *GRN*, but they obtain too few correct edges. The *TPR* of these three methods were 0.2459, 0.2985 and 0.1579, respectively, which were far less than the *TPR* of 0.9424 obtained with the *MEOMI* method. Although the *AUC* of *MEOMI* is slightly lower than that of *PIDC*, the *AUC* and *AUPR* of *MEOMI* were higher than those of the other 8 methods. Overall, *MEOMI* had better learning accuracy when processing a large number of genes.

### 3.3 Experiment results of *E.coli* SOS datasets

**Fig. 8.**
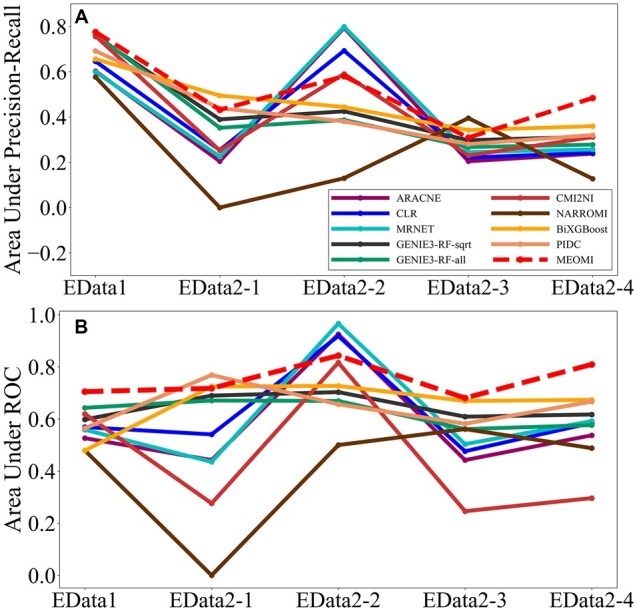
The AUPR and AUROC comparison results on the *E.coli* SOS pathway dataset

The preliminary experimental results showed that the dispersion (*bins*) of the dataset tends to be larger for datasets with a small number of genes and samples. We set *bins* to 8 and 10 for the *E.coli* SOS DNA pathway network and *E.coli* SOS DNA repair network datasets, respectively. In addition, the parameter *λ* was set to 0.016 and 0.003 for the two datasets. We compared the *TP*, *FP*, *TN*, *FN*, *TPR*, *PPV*, *F1*-*score*, *FPR*, *ACC*, *MCC*, *AUPR* and *AUC* of the different methods using the *E.coli* SOS datasets in this experiment. Detailed information can be found in Supplementary Section S6.1. Figure 8 shows the *AUPR* and *AUC* comparison results for the 10 different methods.

**Fig. 9.**
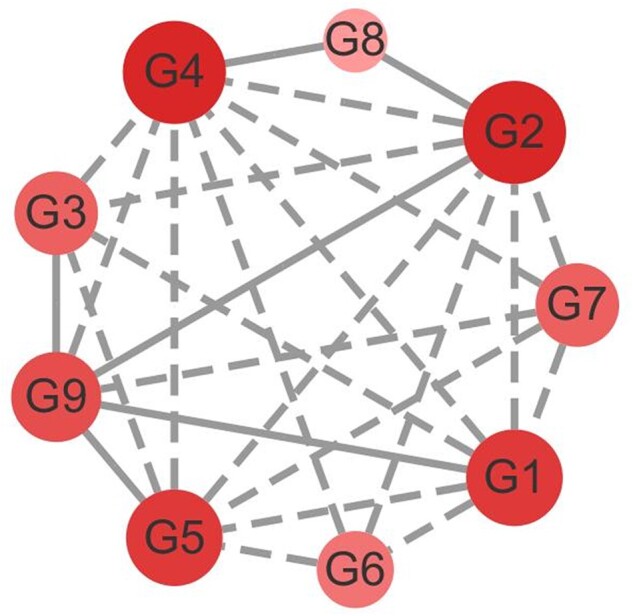
The predicted network of *E.coli* SOS pathway network (EData1)

**Fig. 10.**
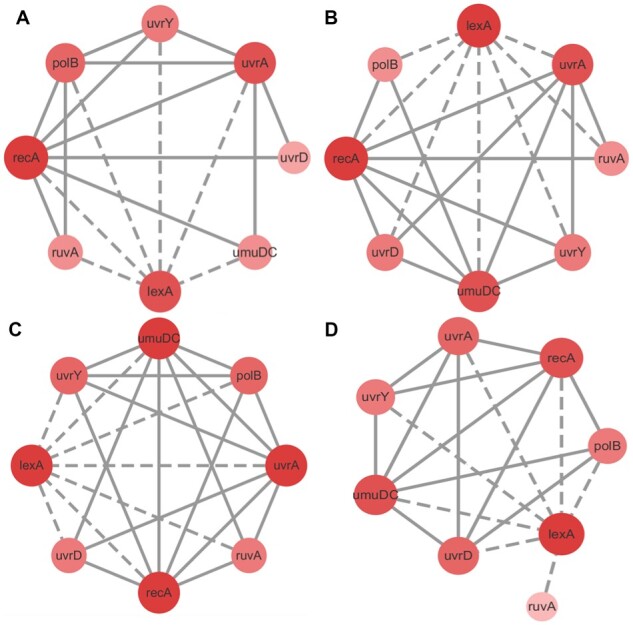
The predicted networks of the *E.coli* SOS DNA repair network. Panels (**A**–**D**) denote the results of EData2-1–EData2-4, respectively

As shown in Figure 8, the *AUC* and *AUPR* of *MEOMI* were better than those of the other methods in the *E.coli* SOS pathway network dataset (EData1). For the *E.coli* SOS DNA repair network dataset (EData2), the *AUC* and *AUPR* of *MEOMI* were essentially better than those of the other methods except for EData2-2. The learning accuracy of *MEOMI* on EData2-2 was lower than that of *ARACNE*, *CLR*, *MRNET*, and there was little difference with *CMI2NI*. The predicted networks of the *E.coli* SOS network (EData1 and EData2) using *MEOMI* are shown in Figures 9 and 10. The solid line represents FP (the incorrectly predicted of edges) and the dotted line represents TP (the correctly predicted edges). It can be seen that the predicted network has relatively high accuracy.

As we known, *lexA* and *recA* are two principle mediators of the SOS response. *MEOMI* can better learn the related edges of *lexA* and *recA* except for EData2-2 dataset. To further analyze why *MEOMI* had a poor learning effect in the EData2-2 dataset, we plotted the distribution of each gene (*polB*, *ruvA*, *uvrY*, *uvrA*, *recA*, *umuDC*, *lexA* and *uvrD*) in the four datasets. Results show that the distribution of *uvrY*, *ruvA*, and *polB* having larger difference with other genes. Further analysis shows that the score of three edges (*lexA*-*polB*, *lexA*-*ruvA*, *lexA*-*uvrY*) were the lowest among all the edges in the standard network. Detailed results and analysis can be seen in Supplementary Section S6.1.

## 4 Discussion

The construction of gene regulatory networks is an important research topic in the field of computational biology. In this work, we combined James–Stein entropy estimation with no prior distribution and Bayes entropy estimation with a Dirichlet prior distribution to calculate the mutual information between genes to obtain an initial gene regulatory network. This was conducive to obtaining more correct edges between genes while avoiding losing as many correct edges as possible. To consider the context information, we optimized the mutual information matrix distribution using the CLR algorithm. Through the above two steps, we obtained an initial gene regulatory network with more correct edges. The mutual information method cannot handle indirect regulatory relationships between genes effectively. The constructed gene network still contained a large number of incorrect regulatory relationships and had a high false-positive rate. We eliminated some indirect regulatory relationships between genes by considering the cumulative distribution of the mutual information. To delete redundant and incorrect edges, we optimized the whole network by calculating the conditional mutual inclusive information between genes under the influence of multiple genes. The initial gene regulatory network was gradually optimized through multiple iterations, until a more accurate gene regulatory network was obtained. In the *MEOMI* method, the parameter *order* has a great influence on the experimental efficiency. We used the dynamic threshold setting method, which results in the value of *order* being set to a small value to improve the efficiency. Experimental results indicate that *MEOMI* can learn more correct edges and has strong applicability on various types of datasets.

The next step of the research mainly includes the following: (i) Because actual gene regulatory networks have a direction, we intend to consider the direction of the gene regulatory relationships to further reduce the number of unnecessary indirect gene regulatory relationships. (ii) It takes a long time to traverse the whole network when deleting the redundant edges. We will further to improve the efficiency and accuracy in the process of traversing the network, including selecting suitable sequential ordering of nodes (Aghdam *et al.*, 2015), selecting effective conditional gene set using the strategies of order independent (Mahmoodi *et al.*, 2021) and heuristic search (Aghdam *et al.*, 2014), etc. (iii) The experimental parameters have a considerable impact on the accuracy and efficiency of the results. Setting appropriate parameters based on the characteristics and distribution of the dataset, thus reducing the running time and simplifying the experimental process, is another issue to be studied in the future.

## Supplementary Material

btac717_Supplementary_Data
