# Supplementary material for: An approach of gene regulatory network construction using mixed entropy optimizing context-related likelihood mutual information
Source: Bioinformatics. 2022 Nov 7;39(1):btac717. doi: 10.1093/bioinformatics/btac717 (PMC9805593; doi:10.1093/bioinformatics/btac717)
Supplement: btac717_Supplementary_Data [file btac717_supplementary_data.zip › btac717_Supplementary_Data/Supplement.docx]

**Supplementary Information**

**1. Framework of MEOMI**

The *MEOMI* algorithm includes the following steps: data discretization, mutual information calculation between genes, mutual information matrix (*MIM*) construction, likelihood optimization of the mutual information matrix distribution and deletion of redundant edges by calculating the conditional mutual inclusive information (*CMI2*). The framework of the *MEOMI* algorithm is shown in Figure S1. Firstly, the dataset was preprocessed and the shrinkage strength *λ* was calculated (Figure S1(A)). Then, the *MIM* was calculated using the hybrid entropy estimation approach (Figure S1(B)). Finally, the redundant edges in the gene regulatory network were deleted (Figure S1(C)). The specific process of *MEOMI* can be seen in Algorithm S1 (section 2).

Figure S1.  The framework of the MEOMI algorithm. (A) Firstly, the continuous gene expression data is discretized to obtain the count vector *X* of gene *G*. It calculates the shrinkage strength λ according to the mean square error MSE of the true probability and the estimated probability. This can help to ensure the minimization of the risk function. (B) On the basis of the shrinkage strength λ, the James-Stein entropy estimation without prior distribution and the Bayes entropy estimation based on the Dirichlet prior distribution are combined to calculate the mutual information between genes, and thus to obtain the mutual information matrix. Then it can obtain the likelihood mutual information scores of gene pairs through standardizing mutual information matrix. (C) It standardizes and merges the mutual information matrices obtained using the two entropy estimators, and obtains the initialized gene regulatory network. On the basis of the path consistency algorithm, it further to filter the edges by setting a dynamic threshold, and thus to obtain the final gene regulatory network.

**2. MEOMI Algorithm**

The specific process of *MEOMI* is shown in Algorithm S1.

| **Algorithm S1.** *MIOMI* algorithm |
| --- |
| **Require:** Gene expression dataset *original_data*, *P* denotes the dispersion of the dataset, *order* is the number of polygenes that control the direct regulation of gene pairs, *λ*_0_ refers to the initial threshold that used to screen the edges in the gene regulatory network  **Ensure:** Inferred gene regulatory network *G*, Weight matrix of gene regulatory network *Gval*  1: **function** MI_Calculate(*original*_*data*, *P*)  2: *data* ← *Discretize*(*original_data*, *P*)  3: /**K* represents the number of bins with a distribution probability greater than 0*/  4: [*x_i1_*, *x*_i2_, ... ,*x_iK_*] ← *Count*(*data*)  5: **for** *i* ← 1 **to** *n* **do**  6: **for** *j* ← 1 **to** *n* **do**  7: /**R*(*λ*) is the risk function of *λ**/  8: *λ* ← *Minimum*(*R*(*λ*))  9: *a* ← n*(1 *- λ*)/K**λ*  10: *θ^JS^*(*x_iK_*) ← *λ*t* + (1 *- λ*)**θ^ML^*(*x_iK_*)  11: *H^JS^*(*X_i_*) ← *Entropy_shrink*(*θ*^JS^(*x_iK_*))  12: *H^Dir^*(*X_i_*) ← *Dirichlet*(*x_i1_*, *x_i2_*, ... , *x_iK_*; *a*)  13: *MIM_ij_* ← *H*(*X_i_*) + *H*(*X_j_*) - *H*(*X_i_*, *X_j_*)  14: **end for**  15: **end for**  16: **return** *MIM*  17: **end function**  18: **function** MI_Optimize(*MIM*)  19: **for** *i* ← 1 **to** *n* **do**  20: **for** *j* ← 1 **to** *n* **do**  21: *Z_i_* ← (*MIM*[*i***n*+*j*] - *avg*[*i*])(*MIM*[*i***n*+*j*] - *avg*[*i*])/*var*[*i*];  22: *Z_score*[*i***n*+*j*] ← *sqrt*(*Z_i_*+*Z_j_*)  23: **end for**  24: **end for**  25: *GVal_0_* ← *Mean*(*Z_score_Dir*, *Z_score_JS*)  26: **return** *GVal_0_*  27: **end function**  28: **function** GRN_Optimize(*Gval_0_*, *λ_0_*, *order0*)  29: *order* ← -1  30: **for** *i* ← 1 **to** *n* **do**  31: **for** *j* ← 1 **to** *n* **do**  32: **if** *order*<=*order0* **then**  33: *order* ← *order* + 1  34: /*Compute *order_Max* of the adjacent genes between *G_i_* and *G_j_*.*/  35: *m* ← $C_{Order\text{-}Max}^{Order}$  36: **for** *s* ← 1 **to** *m* **do**  37: /*Select out *order* genes from these *m* genes*/  38: *B* ← [*B*_1_, ... ,*B_order_* ]  39: *GVal_order_*(*i*, *j*) ← *Max*(*CMI2*(*i*, *j* \| *B*), *GVal_order_*(*i*, *j*))  40: **if** *GVal_order_*(*i*, *j*)<*λ*_0_**m* **then**  41: *G_order_*(*i*, *j*) ← 0  42: **end if**  43: **end for**  44: **else**  45: continue  46: **end if**  47: **end for**  48: **end for**  49: **return** *G*, *GVal*  50: **end function** |

In Algorithm S1, the first part is the function of *MI_Calculate*(*original*_*data*, *P*). The gene expression data is discretized firstly, denoted as *Discretize*(*original_data*, *P*), to obtain the count matrix *Count*(*data*) related to the dispersion *P*. It is expressed as *K* bins, [*x_i1_*, *x*_i2_, ... , *x_iK_*] (steps 1-4). *Minimum*(*R*(*λ*)) refers to minimizing risk function, and it calculates the contraction strength λ corresponding to each *x_ii_*. It calculates the Dirichlet prior *a_i_* according to the correlation of the two parameters (step 8-9). *Entropy_shrink*(*θ^JS^(x_ii_*)) represents the conversion formula of entropy and probability. It calculates the entropy *H^JS^*(*X_i_*) under James-stein shrinkage estimation (step 10-11). Then it calculates the entropy *H^Dir^*(*X_i_*) under Bayesian polynomial ratio estimation. It estimates the entropy *H^Dir^*(*X_i_*) directly according to the empirical distribution of the known dataset. The above process is denoted as *Dirichlet*(*x_i1_*, *x_i2_*, *... , x_iP_*; *a_1_*,*...*, *a_P_*) (step 12). The mutual information *I*(*X_i_*, *X_j_*) of genes *X_i_* and *X_j_* are calculated respectively using the two entropy estimators. It obtains the mutual information matrices *MIM^Dir^* and *MIM^JS^* of the two shrinkage estimators (step 13).

The second part is the function of *MIM_Optimize*(*MIM*), it calculates the *Z*-score of genes *G_i_* and *G_j_*, and thus to obtain the matrices *Z_score_Dir* and *Z_score_JS* (step 19-22). Then it calculates the average of matrices *Z_score_Dir* and *Z_score_JS*, denoted as *GVal_0_*=*Mean*(*Z_score_Dir*, *Z_score_JS*). *GVal_0_* represents the weight matrix of the edges in the network (step 25).

The third part is the function of *GRN_Optimize*(*Gval_0_*, *λ_0_*, *order0*). It traverses all genes and finds the gene pair *G_i_* and *G_j_* whose adjacency matrix of order is not 0 within the range of *order_Max*. If there is an edge between *G_i_* and *G_j_*, it finds all adjacent genes that have edges with *G_i_* and *G_j_*, and records the total number as *order_Max*. The combination number of *order* genes extracted from *order_Max* is denoted as *m*, and the combination of *order* gene combinations is denoted as *B*. Then it calculates *CMI2*(*i*, *j* | *B*) of *G_i_* and *G_j_* corresponding to *order*, and takes the maximum value *GVal_order_*(*i*, *j*) (step 35-39). Then it updates the current threshold according to *m* and the initial threshold *λ*_0_, and deletes the edges lower than the threshold (step 40-41). According to the above steps, the entire adjacency matrix is judged and updated until no edge will be deleted. Then it exits the loop, and obtains the final gene regulatory network *G* and weight matrix *Gval*.

**3. Mutual Information Calculation**

To calculate the mutual information between the genes, the continuous gene expression dataset was first discretized. If the value of a random variable *X* is distributed in an interval [*a*, *b*], the interval can be divided into equidistant subintervals based on the size of the interval. The number of subintervals is denoted as *bin*. The random variable *X* after discretization is shown in Eq. (1).

$$X=[X_{1},\text{ }X_{2},\text{ }X_{3},......,\text{ }X_{n}]^{T}$$

After the discretization operation, *n* variables *X_i_* under the random variable *X* are distributed in *K* bins, where *K* represents the number of bins with a distribution probability greater than 0 (Meyer *et al*., 2008). The index vector corresponding to each bin is *x_i_*, and the index vector corresponding to the random variable *X* is shown in Eq. (2).

$$\chi=[x_{1},x_{2},x_{3},......,x_{K}]^{T}$$

If the random variables corresponding to genes *G_i_* and *G_j_* in the gene expression data are *X_i_* and *X_j_*, and the probability of the corresponding random variables is *θ*, the mutual information of *G_i_* and *G_j_* in the mutual information matrix *MIM* can be calculated using Eq. (3).

$$I(X_{i};X_{j})=H(X_{i})+H(X_{j})-H(X_{i},X_{j})=\sum_{k_{i}\in X_{i}} \sum_{k_{j}\in X_{j}} \theta(x_{k_{i}},x_{k_{j}})\log\left(\frac{\theta(x_{k_{i}},x_{k_{j}})}{\theta(x_{k_{i}})\theta(x_{k_{j}})}\right)$$

Similarly, the conditional mutual information between random variables *X* and *Y* given *Z* can be calculated with Eq. (4).

$$I(X,Y|Z)=\sum_{x\in X,y\in Y,z\in Z} \theta(x,y,z)\log\frac{\theta(x,y|z)}{\theta(x|z)\theta(y|z)}$$

The Shannon entropy of the random variable X corresponding to a single gene is shown in Eq. (5).

$$H(X)=-\sum_{k\in X} \theta(x_{k})\log\theta(x_{k})$$

**4. Evaluation Metrics**

In order to compare the performance of different methods, we use true positive rate (*TPR*), false positive rate (*FPR*), positive predictive value (*PPV*), overall accuracy rate (*ACC*), *F1-score* and Matthews correlation coefficient (*MCC*) to do experiment evaluation, as shown in Eq. (6)-Eq. (11).

$$TPR=TP/(TP+FN)$$

$$FPR=FP/(TN+FP)$$

$$PPV=TP/(TP+FP)$$

$$ACC=(TP+TN)/(TP+FP+TN+FN)$$

$$F1-score=2PPV\times TPR/(PPV+TPR)$$

$$MCC=\frac{TP\times TN-FP\times FN}{\sqrt{(TP+FP)(TP+FN)(TN+FP)(TN+FN)}}$$

*TP* indicates the number of related edges that are correctly judged, *TN* indicates the number of unrelated edges that are correctly judged, *FP* refers to the number of edges that are originally related but are determined to be uncorrelated, *FN* refers to the number of edges that are originally uncorrelated but are determined to be related. F1-socre is the comprehensive judgment of Precision and Recall. Based on the indicators of *TP*, *FP*, *TN* and *FN* through setting different thresholds, we can get *TPR* and *FPR* for drawing the receiver operating characteristic (*ROC*) curve and *TPR* and *PPV* for drawing the Precision-Recall (*PR*) curve. The area under the *ROC* curve (*AUC*) is another metric for comparing different algorithms. For the Precision-Recall (*PR*) curve, it considers *TP* and *FP* comprehensively in the condition of precision. Under the extremely unbalanced data, *PR* may be more suitable than *ROC*. Therefore, we use *PR* as another metric to measure the performance of different algorithms.

**5. Experiment results of DREAM challenge dataset**

We compare *MEOMI* with some representative gene network construction methods, including *GENIE3* (Huynh-Thu *et al*. 2007), *CLR* (Faith *et al*. 2007), *ARECNE* (Margolin *et al*. 2006), *MRNET* (Meyer *et al*. 2007), *CMI2NI* (Zhang *et al*. 2015), *NARROMI* (Zhang *et al*. 2013), *BiXGBoost* (Ruiqing *et al*. 2018) and *PIDC* (Chan *et al*., 2017). Among them, the algorithms of *CLR*, *ARECNE* and *MRNET* are running using the minet package. The implementation code is R language and we use default parameters to do experiments (Meyer *et al.* 2008). The *GENIE3* is implemented using *R* and *BiXGBoost* is implemented using python language. The *PIDC* is implemented using Julia package, and we do the experiment comparison using default parameters. The above three kinds of methods score all the possible edges for every two genes. For the effective comparison, we take the top specific number of gene pairs to do evaluation based on the total number of predicted edges generated by *MEOMI*. In addition, the algorithms of *NARROMI* and *CMI2NI* are implemented using MATLAB, and we use default parameters to do experiments.

**5.1 TPR comparison results of Size50 and Size100 dataset in DREAM3**

Figure S2 shows the *TPR* comparison results of 10 kinds of methods (*GENIE3-RF-sqrt*, *GENIE3-RF-all*, *CLR*, *ARECNE*, *MRNET*, *CMI2NI*, *NARROMI*, *BiXGBoost*, *PIDC*, *MEOMI*) on Size50 and Size100 dataset in DREAM3. We can see that the *TPR* of *CMI2NI*, *NARROMI* and *ARACNE* is lower than other 7 kinds of methods apparently. It means that these 3 kinds of approaches cannot obtain more number of correct edges. Therefore, we will not give the comparison results of *CMI2NI*, *NARROMI* and *ARACNE* in the following sections.


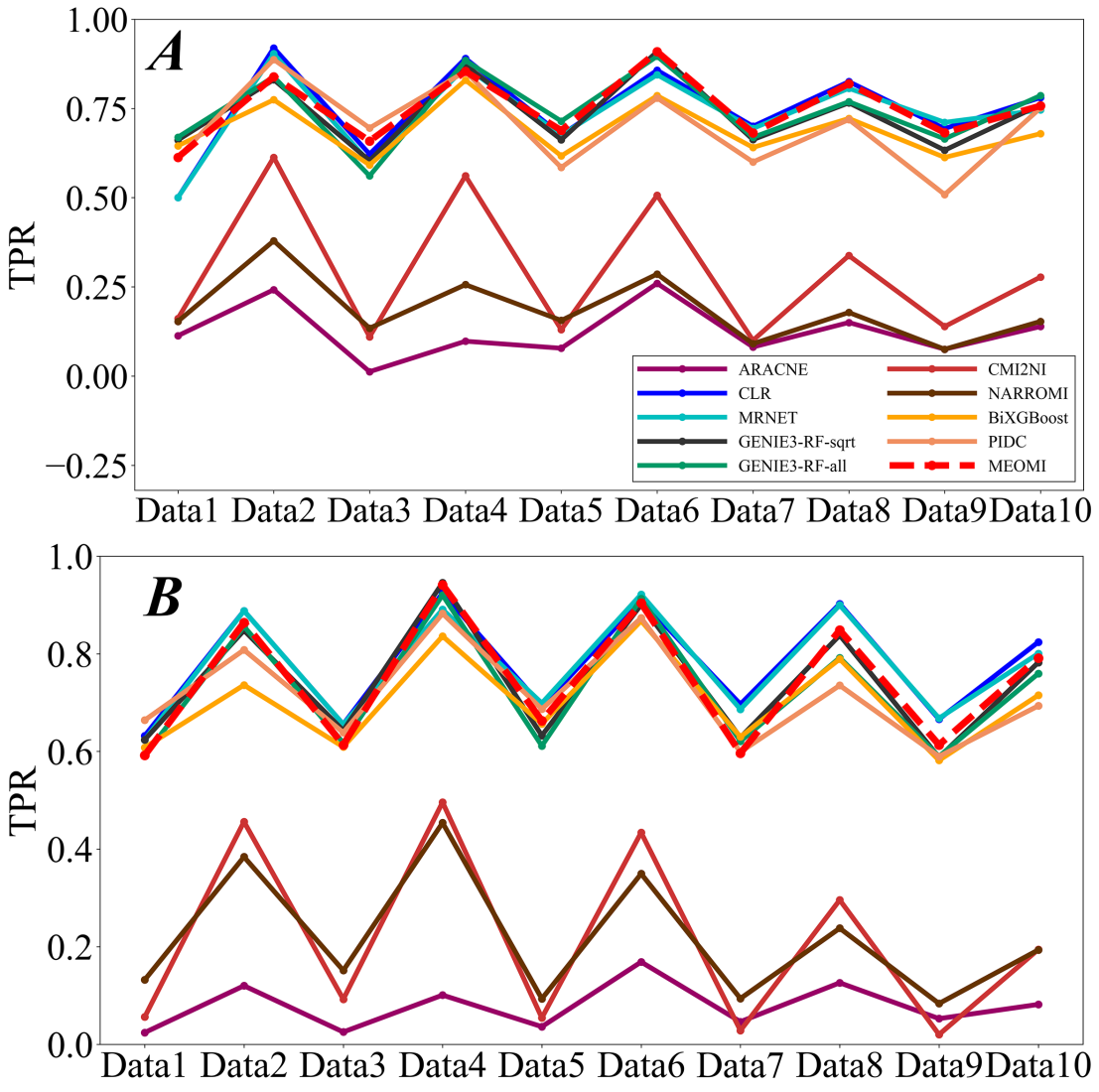


Figure S2. The TPR comparison results of size50 and size100 in DREAM3

Although *CMI2NI* performing better than *MEOMI* on *AUPR* on Size50 dataset, its *TPR* on the 10 datasets were all lower than the *TPR* of *MEOMI*. The detailed *TPR* comparison result between *CMI2NI* and *MEOMI* on the DREAM3 Size50 dataset is shown Figure S3. Similarly, although *CMI2NI* and *NARROMI* performing better than *MEOMI* in term of *AUPR* on Size100 dataset, the corresponding *TPR* on the 10 datasets was lower than *MEOMI*. The detailed *TPR* comparison result of *CMI2NI*, *NARROMI* and *MEOMI* on the DREAM3 Size100 dataset is shown in Figure S4.


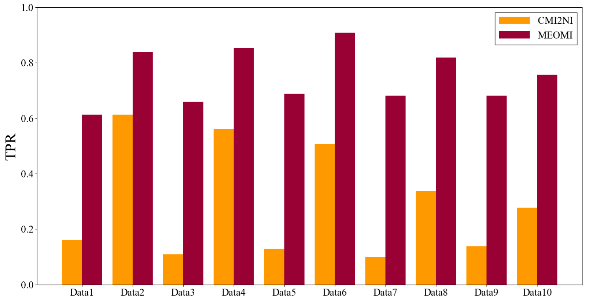


Figure S3. TPR comparison results between CMI2NI and MEOMI of Size50 in DREAM3


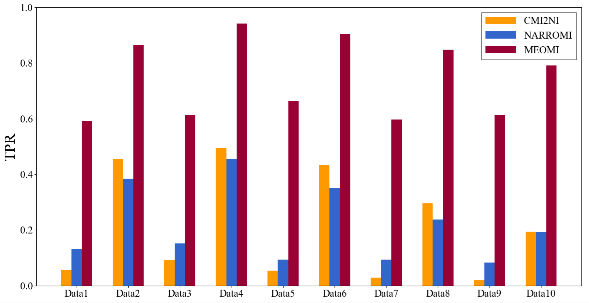


Figure S4. TPR comparison of CMI2NI, NARROMI and MEOMI of Size100 in DREAM3

**5.2 Experiment comparison results of Size50 dataset in DREAM3**

As has been described in section 5.1, the *TPR* of *CMI2NI*, *NARROMI* and *ARACNE* is lower than other 7 kinds of methods apparently. It means that these 3 kinds of approach cannot obtain more number of correct edges, and we only give the comparison results of *GENIE3-RF-sqrt*, *GENIE3-RF-all*, *CLR*, *MRNET* and *BiXGBoost*. Figure S5-Figure S7 elaborate the *F1-score*, *ACC* and *MCC* comparison results of *GENIE3-RF-sqrt*, *GENIE3-RF-all*, *CLR*, *MRNET*, *BiXGBoost*, *PIDC* and *MEOMI* of Size50 dataset in DREAM3.

**
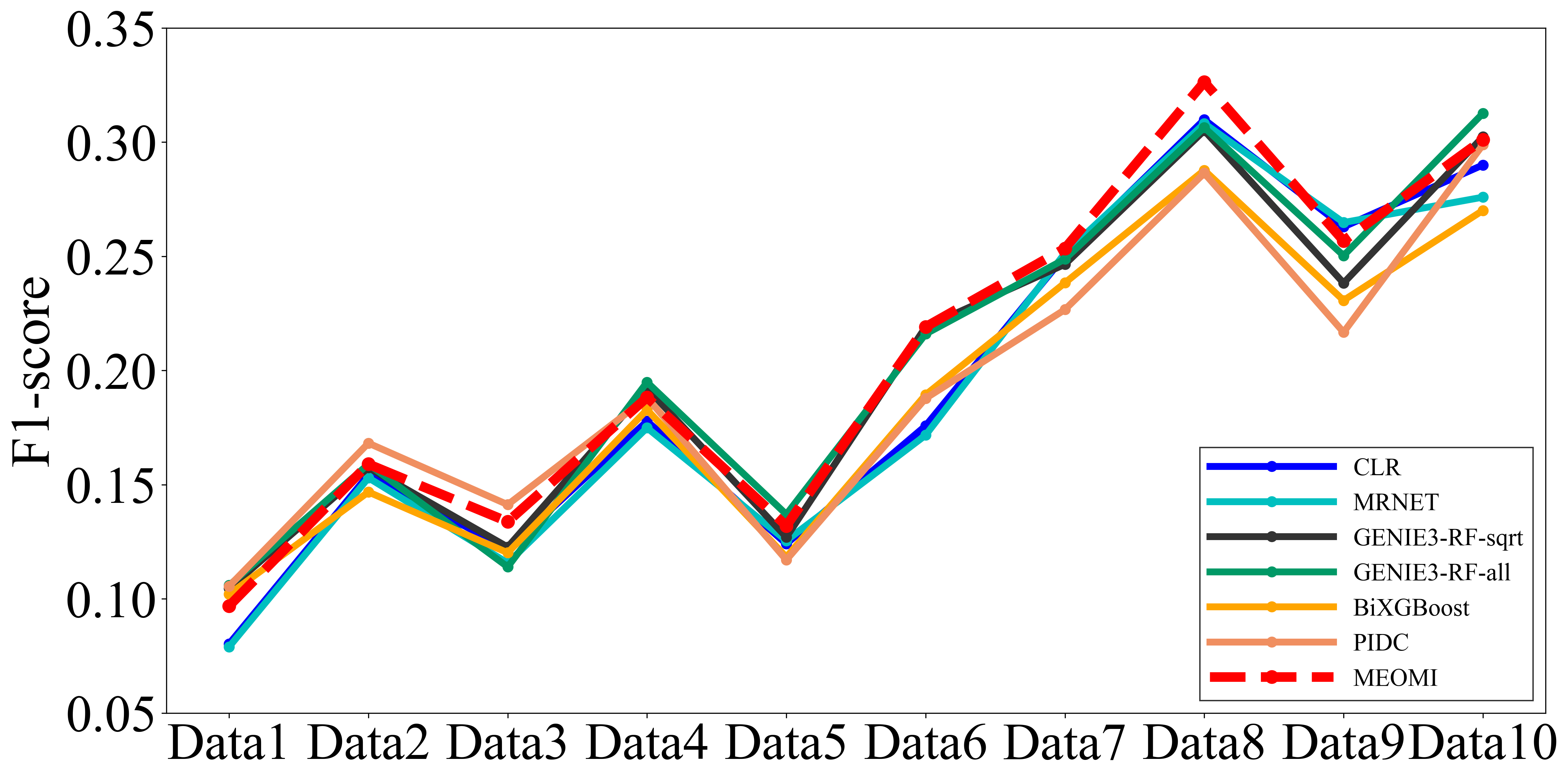
**

Figure S5. The F1-score comparison results of 7 kinds of methods on Size50


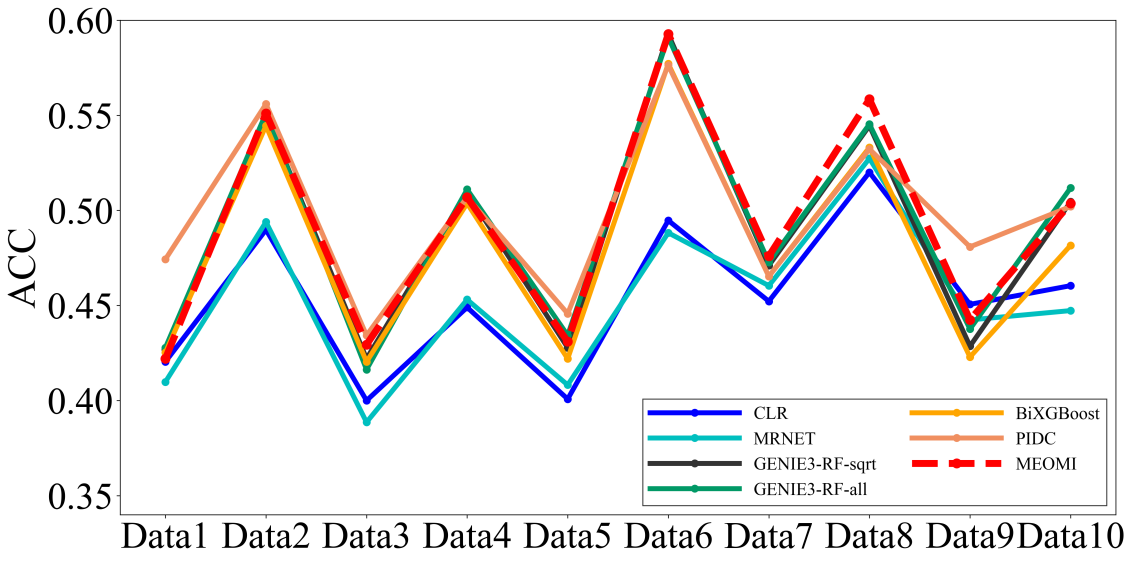


Figure S6. The ACC comparison results of 7 kinds of methods on Size50


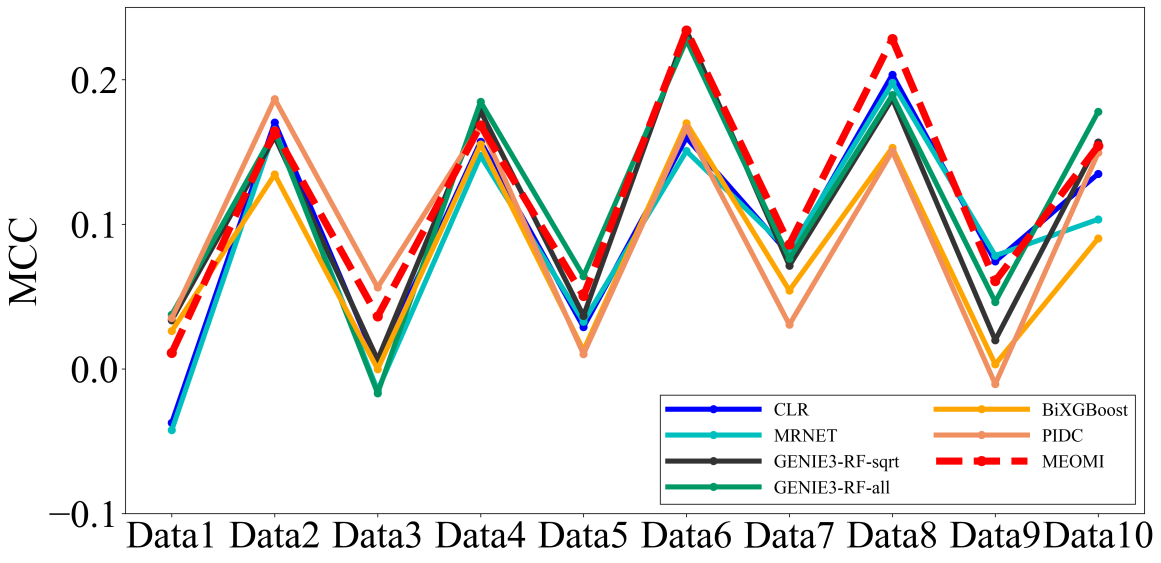


Figure S7. The MCC comparison results of 7 kinds of methods on Size50

From the experiment results of Size50, it can be seen that *MEOMI* method is still superior to *GENIE3-RF-sqrt*, *GENIE3-RF-all*, *CLR*, *MRNET*, *BiXGBoost* and *PIDC* in terms of overall performance while ensuring that there are more number of correct edges in the network.

**5.3 Experiment comparison results of Size100 dataset in DREAM3**

As has been described in section 5.1, the *TPR* of *CMI2NI*, *NARROMI* and *ARACNE* is lower than other 7 kinds of methods apparently. It means that these 3 kinds of approach cannot obtain more number of correct edges, and we only give the comparison results of *GENIE3-RF-sqrt*, *GENIE3-RF-all*, *CLR*, *MRNET*, *BiXGBoost* and *PIDC*. Figure S8-Figure S10 elaborate the *F1-score*, *ACC* and *MCC* comparison results of *GENIE3-RF-sqrt*, *GENIE3-RF-all*, *CLR*, *MRNET*, *BiXGBoost* and *MEOMI* of Size100 dataset in DREAM3.


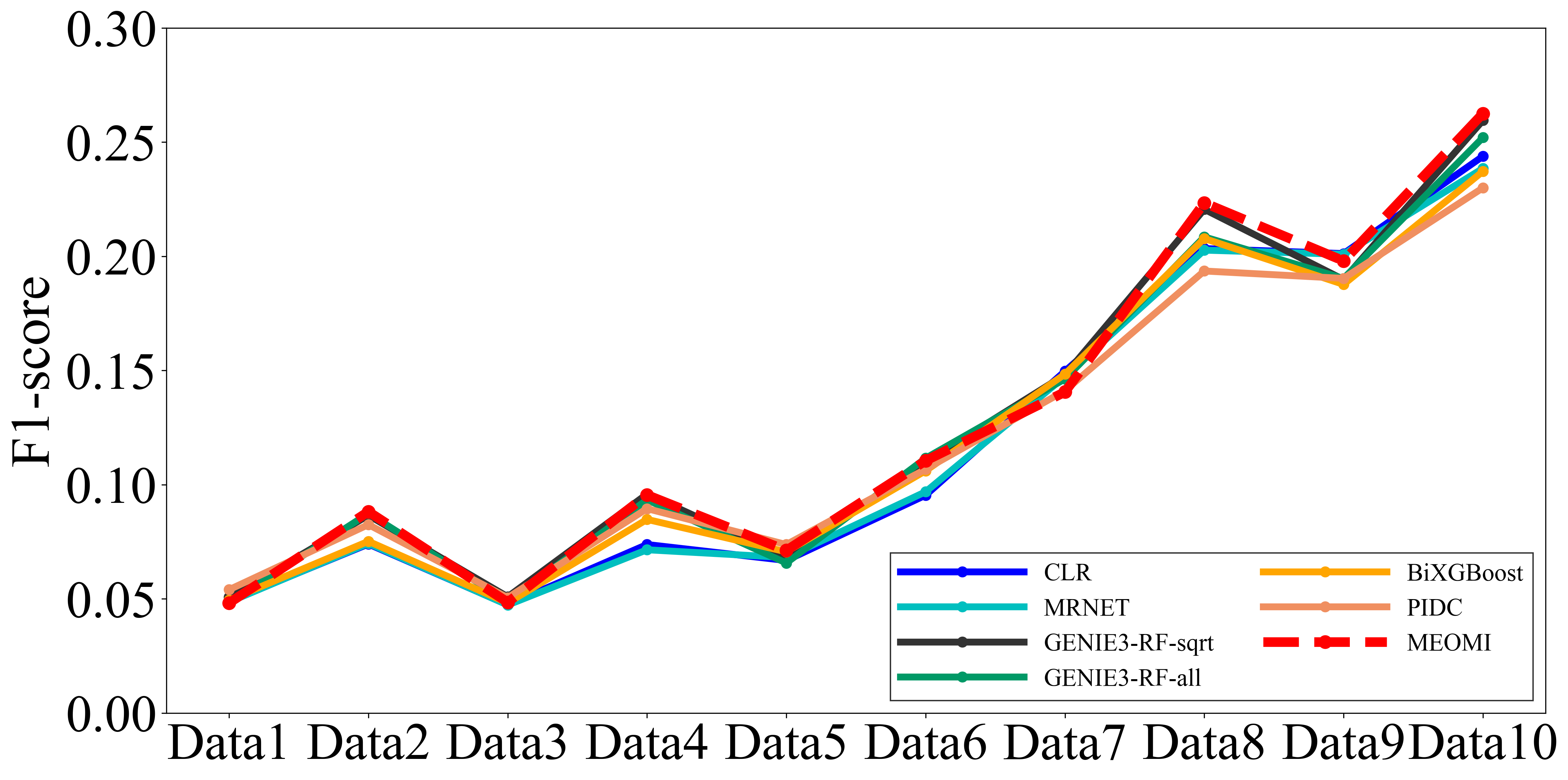


Figure S8. The F1-score comparison results of 7 kinds of methods on Size100


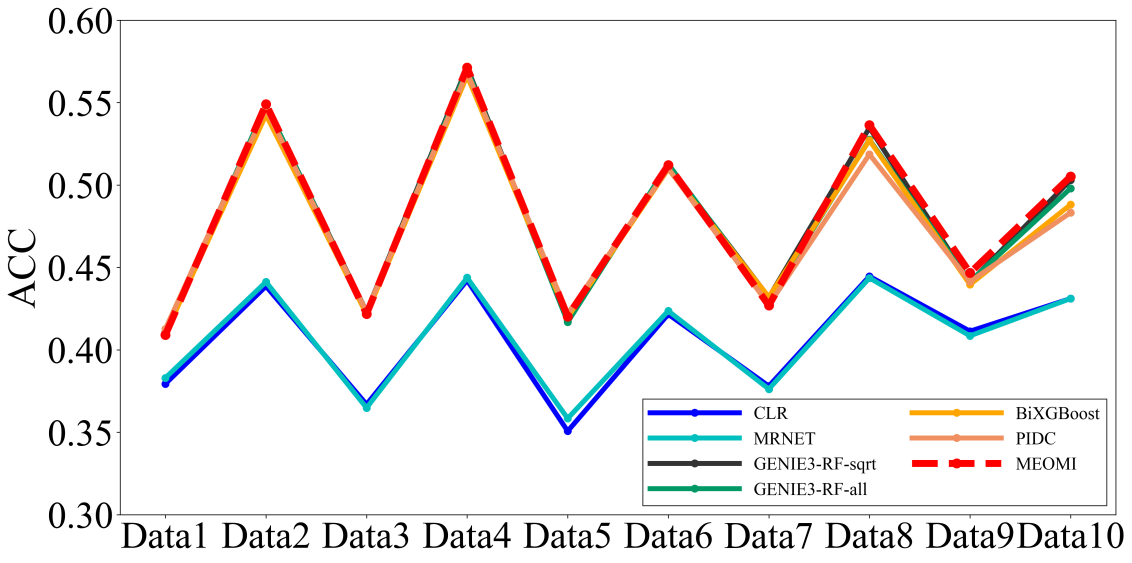


Figure S9. The ACC comparison results of 7 kinds of methods on Size100


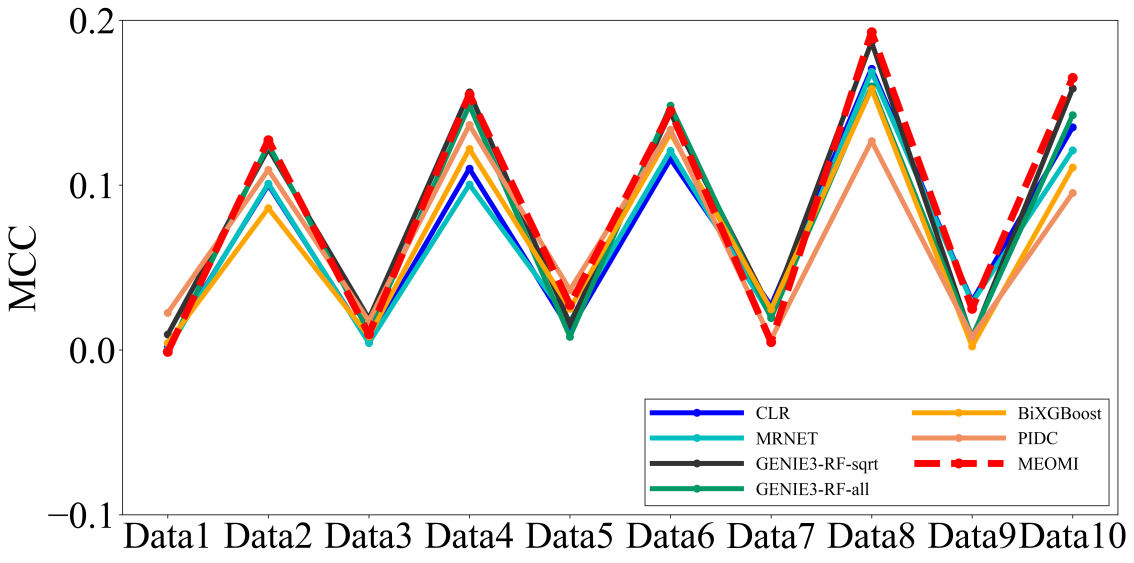


Figure S10. The MCC comparison results of 7 kinds of methods on Size100

From the experiment results of Size100, we can see that *MEOMI* method performing better than *GENIE3-RF-sqrt*, *GENIE3-RF-all*, *CLR*, *MRNET*, *BiXGBoost* and *PIDC* on the whole.

**5.4 Experiment results of Size1643 dataset in DREAM5**

We compare *TP*, *FP*, *TN*, *FN*, *TPR*, *PPV*, *F1-score*, *FPR*, *ACC*, *MCC*, *AUPR* and *AUC* of different methods in the Size1643 dataset. The detailed experiment result information of Size1643 dataset is shown in Table S1.

Table S1. The result of Size1643-net1-expression-datas

| **Method** | **TP** | **FP** | **TPR** | **PPV** | **F1-score** | **FPR** | **ACC** | **MCC** | **AUPR** | **AUC** |
| --- | --- | --- | --- | --- | --- | --- | --- | --- | --- | --- |
| **ARACNE** | 1262 | 4766 | 0.1579 | 0.0080 | 0.2094 | 0.1800 | 0.9809 | 0.1723 | 0.1736 | 0.5752 |
| **CLR** | 7928 | 585374 | 0.9920 | 0.9846 | 0.0134 | 0.0264 | 0.0284 | 0.0069 | 0.1617 | 0.7241 |
| **MRNET** | 7928 | 585502 | 0.9920 | 0.9848 | 0.0134 | 0.0264 | 0.0282 | 0.0068 | 0.1097 | 0.6984 |
| **GENIE3-RF-sqrt** | 4226 | 315964 | 0.5288 | 0.0132 | 0.0258 | 0.5314 | 0.4694 | -0.0006 | 0.1219 | 0.5463 |
| **GENIE3-RF-all** | 4226 | 315964 | 0.5288 | 0.0132 | 0.0258 | 0.5314 | 0.4694 | -0.0006 | 0.1839 | 0.5445 |
| **CMI2NI** | 2386 | 8340 | 0.2985 | 0.2225 | 0.2549 | 0.0140 | 0.9769 | 0.2462 | 0.0697 | 0.6258 |
| **NARROMI** | 1965 | 45545 | 0.2459 | 0.0414 | 0.0708 | 0.0766 | 0.9144 | 0.0719 | 0.1222 | 0.5587 |
| **BiXGBoost** | 4219 | 314464 | 0.5279 | 0.0132 | 0.0258 | 0.5289 | 0.4718 | -0.0002 | 0.1450 | 0.5474 |
| **PIDC** | 7816 | 554960 | 0.9780 | 0.0139 | 0.0274 | 0.9334 | 0.0787 | 0.0205 | 0.2349 | 0.7776 |
| **MEOMI** | 7532 | 555244 | 0.9424 | 0.0134 | 0.0264 | 0.9339 | 0.0777 | 0.0039 | 0.2491 | 0.7463 |

**5.5 Efficiency comparison results in DREAM3 and DREAM5**

In *MEOMI* algorithm, it calculates the mutual information between genes using the hybrid entropy estimation method firstly. Then it will delete redundant edges by calculating the conditional mutual inclusive information (*CMI2*). Multi-step calculation will cost some running time, leading to the efficiency of *MEOMI* will be lower than some kinds of approaches. Table S2 elaborates the running time of all the methods in DREAM3 and DREAM5 datasets.

Table S2. The efficiency comparison in DREAM3 and DREAM5 datasets (seconds)

| **Methods** | **Size50(DREAM3)** | **Size100(DREAM3)** | **Size1643(DREAM5)** |
| --- | --- | --- | --- |
| **ARACNE** | 0.68 | 3.67 | 611.2 |
| **CLR** | 0.62 | 2.36 | 603.52 |
| **MRNET** | 0.64 | 2.38 | 589.64 |
| **GENIE3-RF-sqrt** | 6.95 | 37.04 | 16300 |
| **GENIE3-RF-all** | 24.72 | 247.71 | 159683.92 |
| **CMI2NI** | 1.15 | 7.67 | 987.65 |
| **NARROMI** | 1.33 | 7.22 | 108.91 |
| **BiXGBoost** | 7.7 | 7 | 967 |
| **PIDC** | 0.61 | 0.763 | 2127.92 |
| **MEOMI** | 22.53 | 247.89 | 2669.79 |

Through Table S2, it can be seen that *GENIE3* and *MOMEI* cost more time than other methods apparently. The running time of *PIDC* is less than *MEOMI* apparently for the dataset of Size50(DREAM3) and Size100(DREAM3). But the running time of *PIDC* and *MEOMI* is not much difference for the large dataset of Size1643(DREAM5). The time of *CMI2NI*, *NARROMI* and *BiXGBoost* is more than *ARACNE*, *CLR* and *MRNET*. This is mainly related to the computation complexity of all the algorithms. For *GENIE3*, it mainly uses the tree-based ensemble random forest method to predict the expression pattern of target genes based on the expression patterns of other genes, thus to obtain the gene regulatory relationships. It needs a lot of time to do calculation and constructing the tree, therefore, *GENIE3* uses the most running time compared with other algorithms. Similarly, *MEOMI* includes the steps of mutual information calculation based on James-Stein entropy estimation and Bayes entropy estimation, likelihood optimization of the mutual information matrix distribution and deletion of redundant edges. In order to ensure the accuracy of the final gene regulatory network, it consumes a lot of time to do computation in *MEOMI*. Therefore, *MEOMI* can obtain more accurate gene regulatory network although it takes more running time than other methods.

**5.6 Experiment results of each step in MEOMI**

*MEOMI* mainly includes two steps: constructing initial gene regulatory network based on mixed entropy estimation (Step 1), deleting redundant edges based on conditional mutual inclusive information calculation (Step 2). In this experiment, we randomly select two datasets (Ecoli1-null-mutants, Ecoli2-null-mutants) in DREAM3 to exhibit the concrete results of each step in *MEOMI*. We selected the predicted top 100 edges in the network of all the methods to do comparison, and the experiment results are shown in Table S3 and Table S4. In the tables, *MEOMI*-Step 2 expresses the experimental results of after deleting redundant edges about *MEOMI*. *MEOMI*-Step 1 expresses the experimental results of before deleting redundant edges through referring to the number of correct edges about *MEOMI*-Step 2.

Table S3. The experimental results of two steps on DREAM3-InSilicoSize50-Ecoli1-null-mutants

| **Method** | **TP** | **FP** | **TPR** | **PPV** | **F1-score** | **FPR** | **ACC** | **MCC** | **AUPR** | **AUC** |
| --- | --- | --- | --- | --- | --- | --- | --- | --- | --- | --- |
| **ARACNE** | 16 | 84 | 0.129 | 0.160 | 0.143 | 0.036 | 0.922 | 0.103 | 0.122 | 0.547 |
| **CLR** | 18 | 82 | 0.145 | 0.180 | 0.161 | 0.035 | 0.923 | 0.122 | 0.120 | 0.555 |
| **MRNET** | 20 | 80 | 0.161 | 0.200 | 0.179 | 0.034 | 0.925 | 0.141 | 0.147 | 0.564 |
| **GENIE3-RF-sqrt** | 30 | 70 | 0.242 | 0.300 | 0.268 | 0.030 | 0.933 | 0.235 | 0.218 | 0.606 |
| **GENIE3-RF-all** | 22 | 78 | 0.177 | 0.220 | 0.196 | 0.034 | 0.927 | 0.159 | 0.148 | 0.572 |
| **CMI2NI** | 54 | 46 | 0.435 | 0.540 | 0.482 | 0.020 | 0.953 | 0.461 | 0.455 | 0.709 |
| **NARROMI** | 42 | 58 | 0.339 | 0.420 | 0.375 | 0.025 | 0.943 | 0.348 | 0.383 | 0.659 |
| **BiXGBoost** | 24 | 76 | 0.194 | 0.240 | 0.214 | 0.033 | 0.928 | 0.178 | 0.165 | 0.580 |
| **PIDC** | 14 | 86 | 0.113 | 0.140 | 0.125 | 0.037 | 0.920 | 0.084 | 0.097 | 0.537 |
| **MEOMI-Step1** | 52 | **162** | 0.419 | 0.243 | 0.308 | 0.070 | 0.904 | 0.272 | 0.208 | 0.676 |
| **MEOMI-Step2** | 52 | **48** | 0.419 | 0.520 | 0.464 | 0.021 | 0.951 | 0.442 | 0.406 | 0.700 |

Table S4. The experimental results of two steps on DREAM3-InSilicoSize50-Ecoli2-null-mutants

| **Method** | **TP** | **FP** | **TPR** | **PPV** | **F1-score** | **FPR** | **ACC** | **MCC** | **AUPR** | **AUC** |
| --- | --- | --- | --- | --- | --- | --- | --- | --- | --- | --- |
| **ARACNE** | 10 | 90 | 0.061 | 0.100 | 0.076 | 0.039 | 0.900 | 0.027 | 0.083 | 0.510 |
| **CLR** | 18 | 82 | 0.110 | 0.180 | 0.136 | 0.036 | 0.907 | 0.093 | 0.134 | 0.537 |
| **MRNET** | 12 | 88 | 0.073 | 0.120 | 0.091 | 0.038 | 0.902 | 0.044 | 0.093 | 0.517 |
| **GENIE3-RF-sqrt** | 37 | 63 | 0.226 | 0.370 | 0.280 | 0.028 | 0.922 | 0.250 | 0.288 | 0.600 |
| **GENIE3-RF-all** | 33 | 67 | 0.201 | 0.330 | 0.250 | 0.029 | 0.919 | 0.217 | 0.240 | 0.586 |
| **CMI2NI** | 52 | 48 | 0.317 | 0.520 | 0.394 | 0.021 | 0.935 | 0.374 | 0.400 | 0.649 |
| **NARROMI** | 41 | 59 | 0.250 | 0.410 | 0.311 | 0.026 | 0.926 | 0.283 | 0.330 | 0.614 |
| **BiXGBoost** | 28 | 72 | 0.171 | 0.280 | 0.212 | 0.031 | 0.915 | 0.176 | 0.204 | 0.570 |
| **PIDC** | 14 | 86 | 0.085 | 0.140 | 0.106 | 0.038 | 0.904 | 0.060 | 0.107 | 0.524 |
| **MEOMI-Step1** | 52 | **124** | 0.317 | 0.300 | 0.306 | 0.054 | 0.904 | 0.254 | 0.242 | 0.633 |
| **MEOMI-Step2** | 52 | **48** | 0.317 | 0.520 | 0.394 | 0.021 | 0.935 | 0.374 | 0.379 | 0.648 |

In addition, we selected the datasets with the type of heterozygous in size50 and size100 to do comparison, and the experiment results are shown in Table S5.

Table S5. The experimental results of two steps on the datasets of Size50 and Size100 about MEOMI

|  | **TP** | **FP** | **TPR** | **PPV** | **F1-score** | **FPR** | **ACC** | **MCC** | **AUPR** | **AUC** |
| --- | --- | --- | --- | --- | --- | --- | --- | --- | --- | --- |
| **DREAM3-InSilicoSize50-Ecoli1-heterozygous** | | | | | | | | | | |
| **MEOMI-Step1** | 20 | 240 | 0.1613 | 0.0769 | 0.1042 | 0.1032 | 0.8596 | 0.0414 | 0.0672 | 0.5293 |
| **MEOMI-Step2** | 20 | 80 | 0.1613 | 0.2000 | 0.1786 | 0.0344 | 0.9249 | 0.1406 | 0.1773 | 0.5652 |
| **DREAM3-InSilicoSize50-Ecoli2-heterozygous** | | | | | | | | | | |
| **MEOMI-Step1** | 22 | 170 | 0.1341 | 0.1146 | 0.1236 | 0.0744 | 0.8727 | 0.0556 | 0.0911 | 0.5285 |
| **MEOMI-Step2** | 22 | 78 | 0.1341 | 0.2200 | 0.1667 | 0.0341 | 0.9102 | 0.1263 | 0.1672 | 0.5505 |
| **DREAM3-InSilicoSize50-Yeast1-heterozygous** | | | | | | | | | | |
| **MEOMI-Step1** | 12 | 100 | 0.0779 | 0.1071 | 0.0902 | 0.0436 | 0.9012 | 0.0399 | 0.0905 | 0.5177 |
| **MEOMI-Step2** | 12 | 88 | 0.0779 | 0.1200 | 0.0945 | 0.0383 | 0.9061 | 0.0486 | 0.0958 | 0.5199 |
| **DREAM3-InSilicoSize50-Yeast2-heterozygous** | | | | | | | | | | |
| **MEOMI-Step1** | 20 | 106 | 0.0625 | 0.1587 | 0.0897 | 0.0498 | 0.8343 | 0.0194 | 0.1441 | 0.5062 |
| **MEOMI-Step2** | 20 | 80 | 0.0625 | 0.2000 | 0.0952 | 0.0376 | 0.8449 | 0.0425 | 0.1711 | 0.5127 |
| **DREAM3-InSilicoSize50-Yeast3-heterozygous** | | | | | | | | | | |
| **MEOMI-Step1** | 34 | 152 | 0.0983 | 0.1828 | 0.1278 | 0.0722 | 0.8106 | 0.0342 | 0.1657 | 0.5134 |
| **MEOMI-Step2** | 34 | 66 | 0.0983 | 0.3400 | 0.1525 | 0.0314 | 0.8457 | 0.1177 | 0.2705 | 0.5340 |
| **DREAM3-InSilicoSize100-Ecoli1-heterozygous** | | | | | | | | | | |
| **MEOMI-Step1** | 12 | 298 | 0.0480 | 0.0387 | 0.0429 | 0.0309 | 0.9459 | 0.0154 | 0.0315 | 0.5081 |
| **MEOMI-Step2** | 12 | 88 | 0.0480 | 0.1200 | 0.0686 | 0.0091 | 0.9671 | 0.0610 | 0.0744 | 0.5194 |
| **DREAM3-InSilicoSize100-Ecoli2-heterozygous** | | | | | | | | | | |
| **MEOMI-Step1** | 20 | 446 | 0.0840 | 0.0429 | 0.0568 | 0.0462 | 0.9329 | 0.0274 | 0.0342 | 0.5187 |
| **MEOMI-Step2** | 20 | 80 | 0.0840 | 0.2000 | 0.1183 | 0.0083 | 0.9699 | 0.1160 | 0.1511 | 0.5381 |
| **DREAM3-InSilicoSize100-Yeast1-heterozygous** | | | | | | | | | | |
| **MEOMI-Step1** | 14 | 200 | 0.0422 | 0.0654 | 0.0513 | 0.0209 | 0.9477 | 0.0263 | 0.0509 | 0.5107 |
| **MEOMI-Step2** | 14 | 86 | 0.0422 | 0.1400 | 0.0648 | 0.0090 | 0.9592 | 0.0597 | 0.0895 | 0.5166 |
| **DREAM3-InSilicoSize100-Yeast2-heterozygous** | | | | | | | | | | |
| **MEOMI-Step1** | 20 | 110 | 0.0257 | 0.1538 | 0.0441 | 0.0121 | 0.9123 | 0.0323 | 0.1179 | 0.5068 |
| **MEOMI-Step2** | 20 | 80 | 0.0257 | 0.2000 | 0.0456 | 0.0088 | 0.9154 | 0.0456 | 0.1423 | 0.5085 |
| **DREAM3-InSilicoSize100-Yeast3-heterozygous** | | | | | | | | | | |
| **MEOMI-Step1** | 18 | 112 | 0.0163 | 0.1385 | 0.0292 | 0.0127 | 0.8792 | 0.0100 | 0.1251 | 0.5018 |
| **MEOMI-Step2** | 18 | 82 | 0.0163 | 0.1800 | 0.0300 | 0.0093 | 0.8822 | 0.0221 | 0.1464 | 0.5035 |

Through Table S3-Table S5, it can be seen that *MEOMI* having better experiment results than other 9 kinds of methods on the whole. Note that an initial gene regulation network with large number of redundant edges will be obtained after *MEOMI*-Step 1. Similarly, it can also obtain more number of correct edges in the initial gene regulatory network based on mixed entropy estimation calculation. After deleting large number of redundant edges through the conditional mutual inclusive information calculation (Step 2), *MEOMI* can obtain a more accurate gene regulatory network. Therefore, the two steps in *MEOMI* are all playing an important role in the construction of gene regulatory network with high quality.

**5.7 Experiment results of parameter *order* in MEOMI**

As has been described in section 3.2.1 in the main body, the parameter *order* is the number of polygenes that control the direct regulation of gene pairs in the step of reducing redundant edges. The algorithm will terminate when *order* reaches a certain threshold. The value of *order* has a great impact on the running time of the algorithm, and setting a larger *order* leads to low efficiency of *MEOMI*. In general, we set *order*=2 or *order*=3 in *MEOMI*. In this experiment, we randomly select two datasets (Ecoli1-null-mutants, Ecoli2-null-mutants) in DREAM3 to show the influence of different *orders* on the experimental results. We also selected the predicted top 100 edges in the network of all the methods to do comparison, and the experiment results are shown in Table S6 and Table S7. In the tables, *MEOMI*(*order*: 2) and *MEOMI*(*order*: 3) expresses the experimental results of *order*=2 and *order*=3 when to calculate conditional mutual inclusive information between genes under the influence of multiple genes.

Table S6. The experimental results of *order* on DREAM3-InSilicoSize50-Ecoli1-null-mutants

| **Method** | **TP** | **FP** | **TPR** | **PPV** | **F1-score** | **FPR** | **ACC** | **MCC** | **AUPR** | **AUC** |
| --- | --- | --- | --- | --- | --- | --- | --- | --- | --- | --- |
| **ARACNE** | 16 | 84 | 0.129 | 0.160 | 0.143 | 0.036 | 0.922 | 0.103 | 0.122 | 0.547 |
| **CLR** | 18 | 82 | 0.145 | 0.180 | 0.161 | 0.035 | 0.923 | 0.122 | 0.120 | 0.555 |
| **MRNET** | 20 | 80 | 0.161 | 0.200 | 0.179 | 0.034 | 0.925 | 0.141 | 0.147 | 0.564 |
| **GENIE3-RF-sqrt** | 30 | 70 | 0.242 | 0.300 | 0.268 | 0.030 | 0.933 | 0.235 | 0.218 | 0.606 |
| **GENIE3-RF-all** | 22 | 78 | 0.177 | 0.220 | 0.196 | 0.034 | 0.927 | 0.159 | 0.148 | 0.572 |
| **CMI2NI** | 54 | 46 | 0.435 | 0.540 | 0.482 | 0.020 | 0.953 | 0.461 | 0.455 | 0.709 |
| **NARROMI** | 42 | 58 | 0.339 | 0.420 | 0.375 | 0.025 | 0.943 | 0.348 | 0.383 | 0.659 |
| **BiXGBoost** | 24 | 76 | 0.194 | 0.240 | 0.214 | 0.033 | 0.928 | 0.178 | 0.165 | 0.580 |
| **PIDC** | 14 | 86 | 0.113 | 0.140 | 0.125 | 0.037 | 0.920 | 0.084 | 0.097 | 0.537 |
| **MEOMI(*order*: 2)** | 52 | 48 | 0.419 | 0.520 | 0.464 | 0.021 | 0.951 | 0.442 | 0.406 | 0.700 |
| **MEOMI(*order*: 3)** | 52 | 48 | 0.419 | 0.520 | 0.464 | 0.021 | 0.951 | 0.442 | 0.416 | 0.700 |

Table S7. The experimental results of *order* on DREAM3-InSilicoSize50-Ecoli2-null-mutants

| **Method** | **TP** | **FP** | **TPR** | **PPV** | **F1-score** | **FPR** | **ACC** | **MCC** | **AUPR** | **AUC** |
| --- | --- | --- | --- | --- | --- | --- | --- | --- | --- | --- |
| **ARACNE** | 10 | 90 | 0.061 | 0.100 | 0.076 | 0.039 | 0.900 | 0.027 | 0.083 | 0.510 |
| **CLR** | 18 | 82 | 0.110 | 0.180 | 0.136 | 0.036 | 0.907 | 0.093 | 0.134 | 0.537 |
| **MRNET** | 12 | 88 | 0.073 | 0.120 | 0.091 | 0.038 | 0.902 | 0.044 | 0.093 | 0.517 |
| **GENIE3-RF-sqrt** | 37 | 63 | 0.226 | 0.370 | 0.280 | 0.028 | 0.922 | 0.250 | 0.288 | 0.600 |
| **GENIE3-RF-all** | 33 | 67 | 0.201 | 0.330 | 0.250 | 0.029 | 0.919 | 0.217 | 0.240 | 0.586 |
| **CMI2NI** | 52 | 48 | 0.317 | 0.520 | 0.394 | 0.021 | 0.935 | 0.374 | 0.400 | 0.649 |
| **NARROMI** | 41 | 59 | 0.250 | 0.410 | 0.311 | 0.026 | 0.926 | 0.283 | 0.330 | 0.614 |
| **BiXGBoost** | 28 | 72 | 0.171 | 0.280 | 0.212 | 0.031 | 0.915 | 0.176 | 0.204 | 0.570 |
| **PIDC** | 14 | 86 | 0.085 | 0.140 | 0.106 | 0.038 | 0.904 | 0.060 | 0.107 | 0.524 |
| **MEOMI(*order*: 2)** | 52 | 48 | 0.317 | 0.520 | 0.394 | 0.021 | 0.935 | 0.374 | 0.379 | 0.648 |
| **MEOMI(*order*: 3)** | 46 | 54 | 0.280 | 0.460 | 0.348 | 0.024 | 0.930 | 0.324 | 0.344 | 0.629 |

Through Table S6 and Table S7, we can see that there is not significantly difference between the results of *order*=2 and *order*=3. However, larger value of *order* will cost a lot of running time for *MEOMI*. Therefore, we use *order*=2 to do comparison in our experiment.

**6. Experiment results of Escherichia-coil datasets**

**6.1** **Experiment** **results of E. coli SOS network**

Figure S11-Figure S14 elaborate the *TPR*, *F1-score*, *ACC* and *MCC* comparison results of *GENIE3-RF-sqrt*, *GENIE3-RF-all*, *CLR*, *ARECNE*, *MRNET*, *CMI2NI*, *NARROMI*, *BiXGBoost*, *PIDC* and *MEOMI* of E. coli SOS network (EData1~EData2).

**
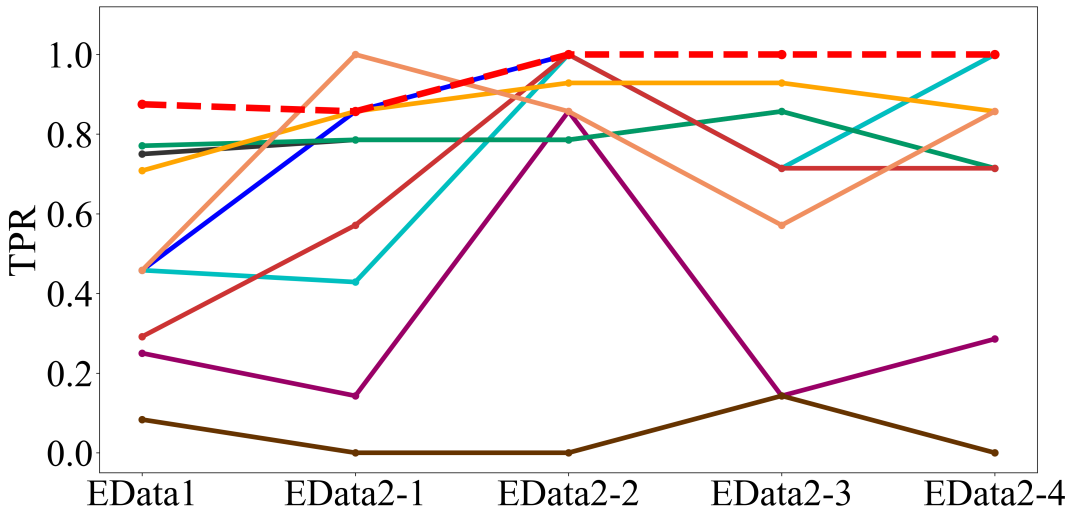
**

Figure S11. The TPR comparison results of E. coli SOS network

**
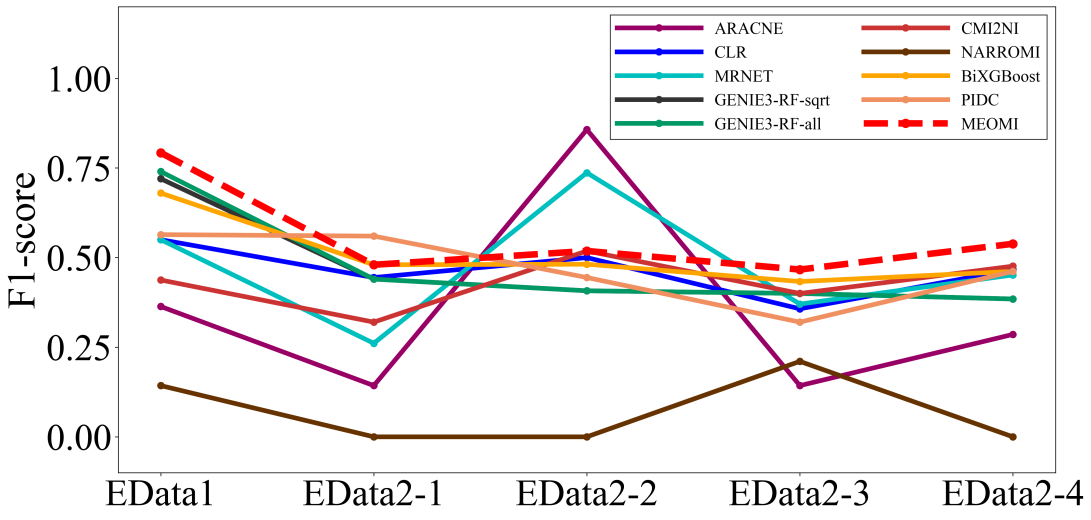
**

Figure S12. The F1-score comparison results of E. coli SOS network


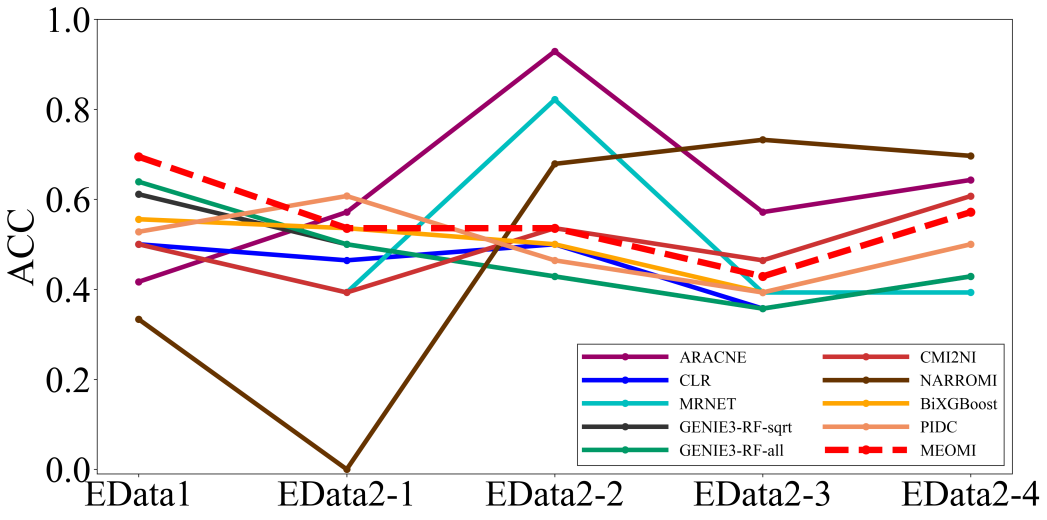


Figure S13. The ACC comparison results of E. coli SOS network

**
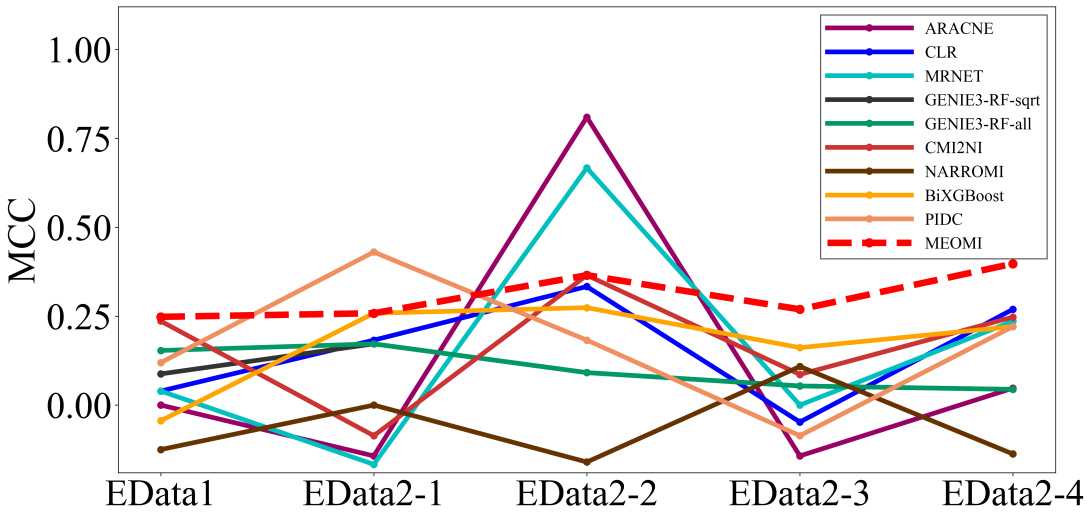
**

Figure S14. The MCC comparison results of E. coli SOS network

From the experiment results, we can see that the *TPR*, *F1-score*, *ACC* and *MCC* of *MEOMI* are better than other 9 kinds of methods on E. coli SOS pathway network dataset (EData1). For the E. coli SOS DNA repair network dataset (EData2), the *TPR*, *F1-score*, *ACC* and *MCC* of *MEOMI* are basically better than other methods except for EData2-2. The reason for the poor performance of *MEOMI* in EData2-2 may be related to the data distribution, as shown in Figure S15.


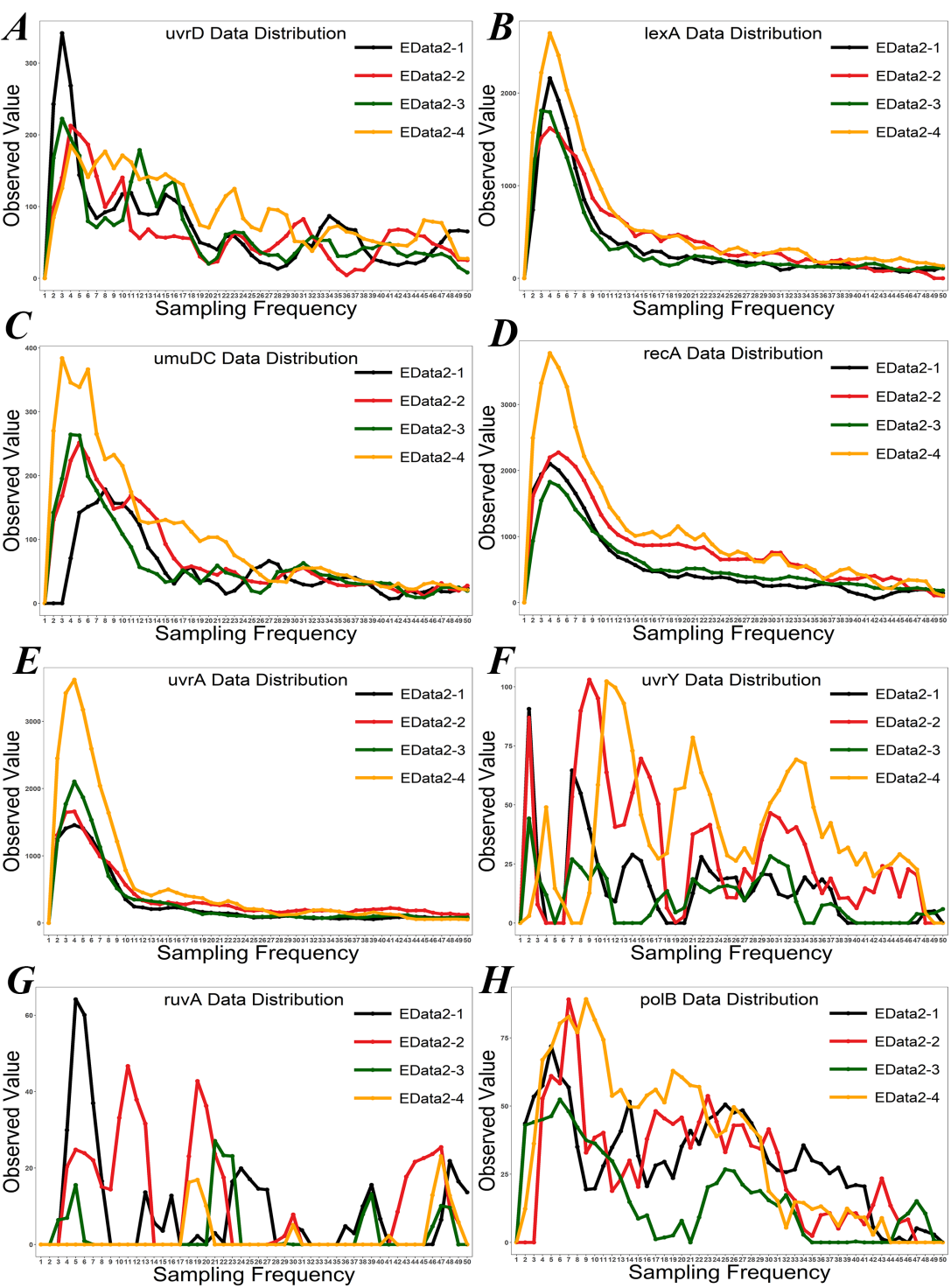


Figure S15. Distribution of 8 genes in the different datasets of the *E. coli* SOS DNA repair network

In Figure S15, the three genes with the largest differences in the distribution of the 4 datasets were *uvrY*, *ruvA*, and *polB*. After further analysis of the constructed gene network of *MEOMI*, we found that the scores of the edges related to these three genes were the lowest among all the edges in the standard network, as shown in Table S8. Therefore, we concluded that the poor *AUC* and *AUPR* performance of the *MEOMI* method in EData2-2 may be related to the data distribution.

Table S8. Prediction scores of gene pairs in the standard network of EData2-2

| **Gene1** | **Gene2** | **Prediction Score** |
| --- | --- | --- |
| lexA | recA | 1 |
| lexA | uvrA | 0.95 |
| lexA | umuDC | 0.9 |
| lexA | uvrD | 0.8 |
| lexA | **polB** | **0.5** |
| lexA | **ruvA** | **0.4** |
| lexA | **uvrY** | **0.25** |

**6.2 Experiment results of Escherichia-coil community network**

The *Escherichia coli* community network dataset contains 4297 genes, including 175 transcription factors and 4564 regulatory edges. The parameters were set to *λ*=0.0001, and *order*=4. We compared the *TP*, *FP*, *TN*, *FN*, *TPR*, *PPV*, *F1-score*, *FPR*, *ACC*, *MCC*, *AUPR*, and *AUC* of the different methods in the *E. coli* community network. Some experimental results are shown in Table S9.

Table S9. The experimental results of the E. coli community network

| **Method** | **TP** | **FP** | **F1-score** | **AUPR** | **AUC** |
| --- | --- | --- | --- | --- | --- |
| **ARACNE** | 768 | 25874 | 0.0463 | 0.0285 | 0.5505 |
| **CLR** | 6456 | 1453452 | 0.0088 | 0.0331 | 0.6993 |
| **MRNET** | 6456 | 1453432 | 0.0088 | 0.0211 | 0.671 |
| **GENIE3-RF-sqrt** | 3384 | 748416 | 0.0089 | 0.0182 | 0.5784 |
| **GENIE3-RF-all** | 3384 | 748416 | 0.0089 | 0.0184 | 0.5701 |
| **CMI2NI** | 2386 | 331926 | 0.014 | 0.0022 | 0.5733 |
| **NARROMI** | 669 | 52103 | 0.0226 | 0.015 | 0.5335 |
| **BiXGBoost** | 3374 | 743961 | 0.009 | 0.0197 | 0.5458 |
| **PIDC** | 6396 | 1422080 | 0.0089 | 0.0463 | 0.6169 |
| **MEOMI** | 6198 | 1422278 | 0.0086 | 0.0208 | 0.6364 |

As shown in Table S8, the *AUC* and *AUPR* of *MEOMI* were slightly lower than those of *CLR* and *MRNET*, but higher than those of *GENIE3*, *CMI2NI*, *NARROMI* and *BiXGBoost*. The *MEOMI*, *CLR* and *MRNET* methods construct gene regulatory networks based on mutual information theory. Although *MEOMI* was slightly inferior to *CLR* and *MRNET* on some parameters, *MEOMI* can still reduce *FP* while ensuring that *TP* does not change considerably. This indicates that *MEOMI* can delete more false-positive edges, effectively reducing the number of unnecessary indirect gene regulatory relationships. In addition, we can see that the *F1-score* of *MEOMI* was lower than those of *ARACNE* and *NARROMI*, and was similar to those of the other methods. However, the *TP* of *ARACNE* and *NARROMI* were far less than that of *MEOMI*, indicating that these two methods cannot obtain more number of correct edges. It also can be seen that the learning effect of *PIDC* and *MEOMI* is about same. The *F1-score* and *AUPR* of *PIDC* is slightly larger than *MEOMI*, but the *AUC* of *PIDC* is slightly smaller than *MEOMI*. Overall, *MEOMI* still has advantages in processing real *E. coli* datasets with a large number of genes. The detailed experiment result of Escherichia-coil community network is shown in Table S10.

Table S10. The result of Escherichia-coil community network

| **Method** | **TP** | **FP** | **TN** | **FN** | **TPR** | **PPV** | **F1-score** | **FPR** | **ACC** | **MCC** | **AUPR** | **AUC** |
| --- | --- | --- | --- | --- | --- | --- | --- | --- | --- | --- | --- | --- |
| **ARACNE** | 768 | 25874 | 1440768 | 5740 | 0.1180 | 0.0288 | 0.0463 | 0.0176 | 0.9785 | 0.0499 | 0.0285 | 0.5505 |
| **CLR** | 6456 | 1453452 | 13190 | 52 | 0.9920 | 0.0044 | 0.0088 | 0.9910 | 0.0133 | 0.0007 | 0.0331 | 0.6993 |
| **MRNET** | 6456 | 1453432 | 13210 | 52 | 0.9920 | 0.0044 | 0.0088 | 0.9910 | 0.0133 | 0.0007 | 0.0211 | 0.6710 |
| **GENIE3-RF-sqrt** | 3384 | 748416 | 718226 | 3124 | 0.5200 | 0.0045 | 0.0089 | 0.5103 | 0.4898 | 0.0013 | 0.0182 | 0.5784 |
| **GENIE3-RF-all** | 3384 | 748416 | 718226 | 3124 | 0.5200 | 0.0045 | 0.0089 | 0.5103 | 0.4898 | 0.0013 | 0.0184 | 0.5701 |
| **CMI2NI** | 2386 | 331926 | 1134716 | 4122 | 0.3666 | 0.0071 | 0.0140 | 0.2263 | 0.7719 | 0.0222 | 0.0022 | 0.5733 |
| **NARROMI** | 669 | 52103 | 1414539 | 5839 | 0.1028 | 0.0127 | 0.0226 | 0.0355 | 0.9607 | 0.0240 | 0.0150 | 0.5335 |
| **BiXGBoost** | 3374 | 743961 | 722681 | 3134 | 0.5184 | 0.0045 | 0.0090 | 0.5073 | 0.4929 | 0.0015 | 0.0197 | 0.5458 |
| **PIDC** | 6396 | 1422080 | 44562 | 112 | 0.9828 | 0.0045 | 0.0089 | 0.9696 | 0.0346 | 0.0051 | 0.0463 | 0.6169 |
| **MEOMI** | 6200 | 1421720 | 44922 | 308 | 0.9527 | 0.0043 | 0.0086 | 0.9694 | 0.0347 | 0.0064 | 0.0209 | 0.6380 |

**7. Experiment Comparisons of the Human Datasets**

We also performed experiment validation using the following two human datasets: gonadal sex determination (GSD) and human mature hepatocytes (hHEP) (Pratapa *et al*., 2020). The GSD dataset included 19 genes, 79 edges and 2000 samples. The GSD datasets with the dropout rate *q*=0 and *q*=50 are used to do experiment comparison. For the hHEP dataset, there are 11515 genes and 425 samples. We selected the top 100, 200, 500 most variable genes to do experiment comparison, and there are 9, 17, 33 TF genes respectively in each datasets. Same to (Pratapa *et al*., 2020), we used the standard network that is obtained from the STRING database to do validation and analysis.

**7.1 Experiment Results of the Gonadal Sex Determination (GSD)**

The gonadal sex determination (GSD) dataset included 19 genes and 2000 samples. We compared different methods in the gonadal sex determination (GSD) dataset with the dropout rate *q*=0 and *q*=50. Detailed information can be found in the Supplementary materials (Results of human datasets.xlsx). Figure S16 shows the *TPR*, *PPV*, *F1-score*, *FPR*, *ACC*, *MCC*, *AUPR*, and *AUC* comparison results of the GSD (drop rate=0) and GSD (drop rate=50) datasets.


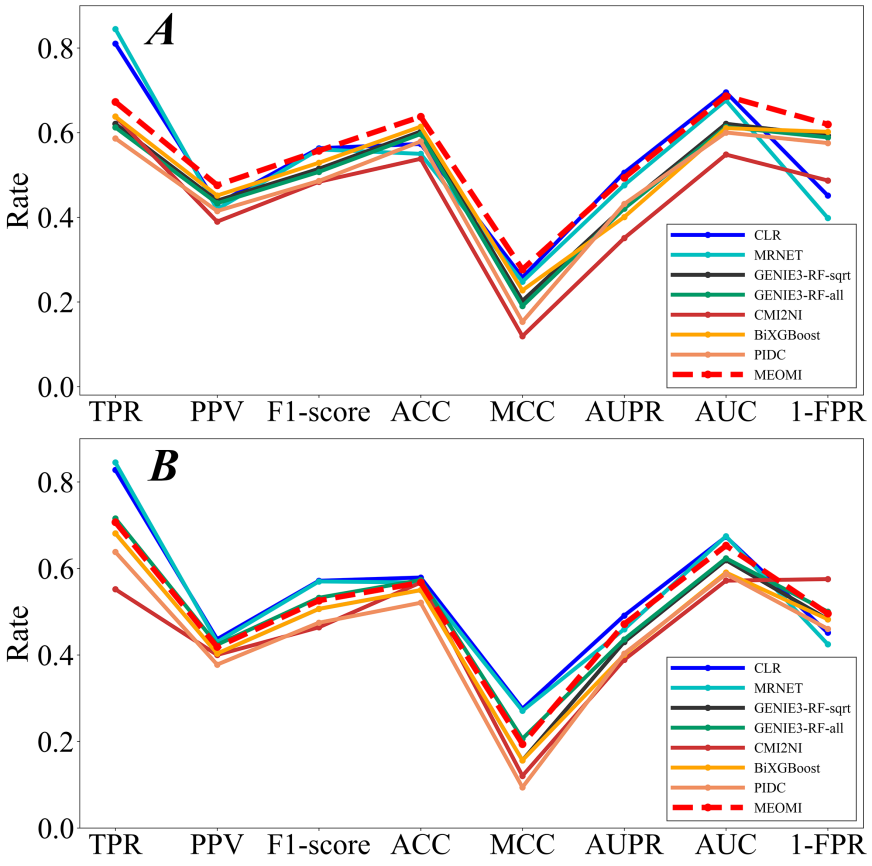


Figure S16. Experiment results on the gonadal sex determination (GSD) dataset.

It should be noted that the *TPR* of *ARACNE* and *NARROMI* is far lower than other 8 kinds of methods apparently. It means that *ARACNE* and *NARROMI* cannot obtain more number of correct edges. Therefore, we will not give the comparison results of the two approaches in this experiment. As shown in Figure S16, the performance of *MEOMI* is better than *GENIE3-RF-sqrt*, *GENIE3-RF-all*, *CMI2NI*, *BiXGBoost* and *PIDC* on the whole. Although the *TPR* of *MEOMI* is lower than that of *CLR* and *MRNET*, the *FPR* of the two methods is far larger than *MEOMI* apparently.

**7.2 Experiment Results of the Human Mature Hepatocytes (hHEP)**

The human mature hepatocytes (hHEP) dataset included 11515 genes and 425 samples. We selected the top 100, 200, 500 most variable genes related to 9, 17, 33 TF genes respectively to perform experimental comparison. We compared different methods in the human mature hepatocytes (hHEP) datasets, and detailed information can be found in the Supplementary materials (Results of human datasets.xlsx). Figure S17 shows the *TPR*, *PPV*, *F1-score*, *FPR*, *ACC*, *MCC*, *AUPR* and *AUC* comparison results of the hHEP (top 100), hHEP (top 200) and hHEP (top 500) datasets.


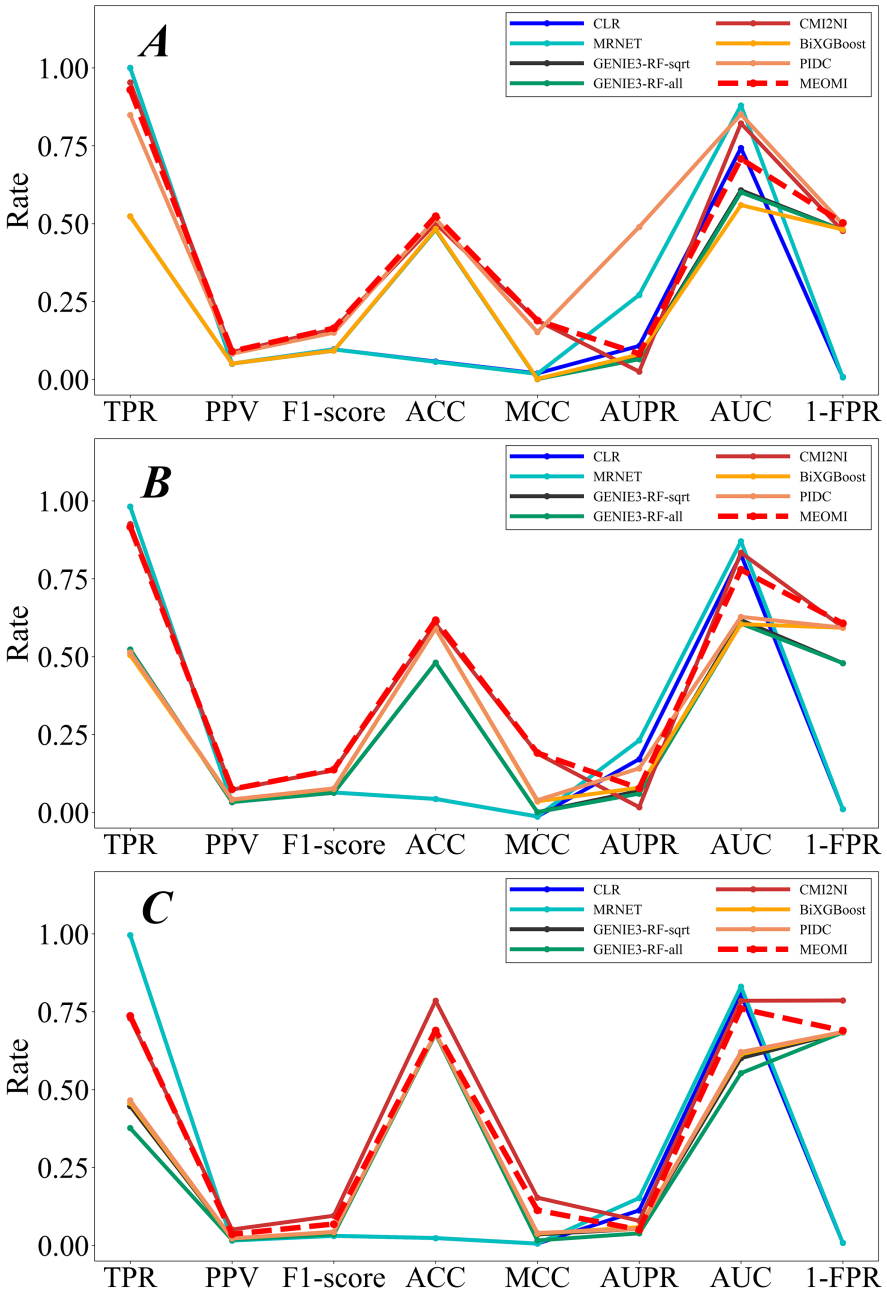


Figure S17.  Experiment results on the human mature hepatocytes (hHEP) dataset.

Similarly, the *TPR* of *ARACNE* and *NARROMI* is far lower than other 8 kinds of methods apparently, we will not give the comparison results of the two approaches in this experiment. Through the experiment results, we can see that the performance of *MEOMI* and *CMI2NI* are better than other 5 kinds of methods on the whole. Although the *TPR* of *MEOMI* is lower than *MRNET* on the hHEP (top 500) dataset, the *FPR* of *MRNET* is far larger than *MEOMI* apparently. The *FPR* of *CLR* and *MRNET* is higher than other 6 kinds of algorithms on the three datasets. Overall, we can see that *MEOMI* has better learning performance than other algorithms when to process more number of genes.

**References**

Chan T E, Stumpf M P H, Babtie A C. Gene regulatory network inference from single-cell data using multivariate information measures. Cell systems 2017; 5(3):251-267.

Daub CO, Steuer R, Selbig J, et al. Estimating mutual information using B-spline functions--an improved similarity measure for analysing gene expression data. BMC Bioinformatics 2004; 5:118.

Huynh-Thu VA, Irrthum A, Wehenkel L, et al. Inferring regulatory networks from expression data using tree-based methods. PLoS One 2010; 5:e12776.

Pratapa,A. et al. Benchmarking algorithms for gene regulatory network inference from single-cell transcriptomic data. Nature Methods 2020; 17(2), 1-8.

Margolin AA, Nemenman I, Basso K, et al. ARACNE: an algorithm for the reconstruction of gene regulatory networks in a mammalian cellular context. BMC Bioinformatics 2006; 7:S7.

Meyer PE, Lafitte F, Bontempi G. minet: A R/Bioconductor Package for Inferring Large Transcriptional Networks Using Mutual Information. BMC Bioinformatics 2008; 9:1-10.

Meyer PE, Kontos K, Lafitte F, et al. Information-theoretic inference of large transcriptional regulatory networks. EURASIP Journal on Bioinformatics and Systems Biology 2007; 2007:79879.

Zhang X, Zhao J, Hao JK, et al. Conditional mutual inclusive information enables accurate quantification of associations in gene regulatory networks. Nucleic Acids Research 2015; 43:e31.

Zhang X, Liu K, Liu ZP, et al. NARROMI: a noise and redundancy reduction technique improves accuracy of gene regulatory network inference. Bioinformatics 2013; 29:106-113.
